# Postnatal β-cell maturation is associated with islet-specific microRNA changes induced by nutrient shifts at weaning

**DOI:** 10.1038/ncomms9084

**Published:** 2015-09-02

**Authors:** Cécile Jacovetti, Scot J. Matkovich, Adriana Rodriguez-Trejo, Claudiane Guay, Romano Regazzi

**Affiliations:** 1 Department of Fundamental Neurosciences, University of Lausanne, Lausanne 1005, Switzerland; 2 Department of Internal Medicine, Center for Pharmacogenomics, Washington University School of Medicine, St Louis, Missouri 63110, USA

## Abstract

Glucose-induced insulin secretion is an essential function of pancreatic β-cells that is partially lost in individuals affected by Type 2 diabetes. This unique property of β-cells is acquired through a poorly understood postnatal maturation process involving major modifications in gene expression programs. Here we show that β-cell maturation is associated with changes in microRNA expression induced by the nutritional transition that occurs at weaning. When mimicked in newborn islet cells, modifications in the level of specific microRNAs result in a switch in the expression of metabolic enzymes and cause the acquisition of glucose-induced insulin release. Our data suggest microRNAs have a central role in postnatal β-cell maturation and in the determination of adult functional β-cell mass. A better understanding of the events governing β-cell maturation may help understand why some individuals are predisposed to developing diabetes and could lead to new strategies for the treatment of this common metabolic disease.

## Abstract (web summary)

Pancreatic β-cells are less responsive to changes in glucose concentration in newborn than in adult rats. Here, the authors show that functional β-cell maturation is associated with changes in miRNA expression induced by nutritional shifts at the suckling-to-weaning transition.

## Graphical abstract

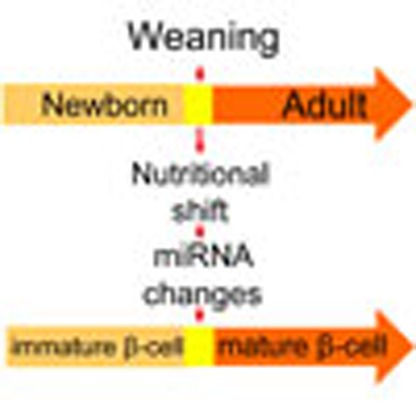

Pancreatic β-cells are the key cell type governing blood glucose homeostasis thanks to their ability to sense changes in nutrient levels and their capacity to adapt the amount of insulin they secrete to match metabolic needs^1^,^2^. β-cell glucose responsiveness is achieved through tight coupling of insulin exocytosis with glycolysis and mitochondrial metabolism^1^. These unique β-cell properties are acquired during a postnatal maturation process. Indeed, in newborn infants plasma insulin levels are increased by amino acid administration but glucose infusion is ineffective in stimulating insulin release^3^,^4^. Numerous studies in rodents have confirmed the absence of glucose-stimulated insulin secretion in newborn β-cells, despite normal insulin content and appropriate ion channel activities^5^,^6^,^7^,^8^,^9^. The immature newborn β-cell phenotype is linked to the presence of strikingly low levels of most glycolytic enzymes and mitochondrial shuttles^10^,^11^,^12^ and, concomitantly, to the expression of the anaerobic glycolytic enzymes Mct1 and Ldha, which are almost absent in mature β-cells^13^.

Another feature of newborn β-cells is their strong replicative potential that allows a significant postnatal β-cell mass expansion^14^. In humans, the greatest proliferation rate is observed before 2 years of age, and after the age of 5 years the mass of β-cells remains relatively constant^15^,^16^. Thus, the neonatal proliferative wave is critical for achieving an appropriate adult β-cell mass and differences in the magnitude of this effect are likely to contribute to inter-individual diabetes susceptibility^17^,^18^.

The suckling–weaning transition is associated with a drastic nutritional shift in which fat-enriched maternal milk is replaced by a carbohydrate-rich diet. This requires extensive and coordinated metabolic adaptations to maintain energy homeostasis^19^,^20^, potentially affecting β-cells. Indeed, glucose is vital for postnatal β-cell development and diet composition has been suggested to influence postnatal β-cell differentiation^21^,^22^,^23^,^24^. However, the contribution of weaning itself to the acquisition of the mature β-cell phenotype and the mechanisms potentially linking the two events remain to be established.

MicroRNAs (miRNAs) are translational repressors that play key roles in the control of β-cell activities and in diabetes pathogenesis^25^,^26^. Deletion of Dicer1, the enzyme required for miRNA processing, in Pdx1-expressing cells results in pancreatic agenesis, while its deletion in insulin-producing cells causes impaired glucose homeostasis and adult diabetes onset^27^,^28^,^29^,^30^. Notably, the absence of Dicer1 in Ngn3-expressing cells does not perturb endocrine cell specification during fetal development but leads to the loss of β-cells and severe metabolic disturbances during the postnatal period^31^. Taken together, these observations point to a critical role for miRNAs in β-cell differentiation.

The aim of this study was to perform a systematic analysis of miRNA expression changes during postnatal β-cell maturation and to assess their contribution to the acquisition of a functionally mature phenotype. Our data demonstrate that the changes in miRNA expression and the maturation of newborn β-cells are largely driven by the nutritional transition that occurs at weaning. Identification of key miRNAs involved in β-cell maturation will help to design therapeutic strategies based on the engineering of functionally competent insulin-secreting cells and will shed new light on possible causes of individual diabetes susceptibility.

## Results

### Phenotypic properties of newborn β-cells

**Figure 1:**
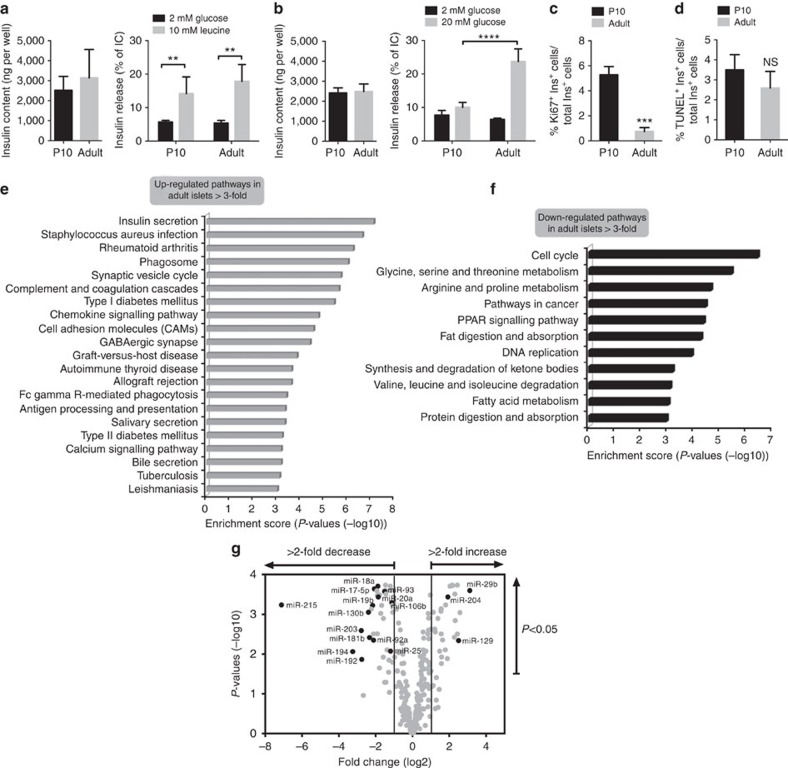
Phenotypic and transcriptomic differences between 10-day-old and adult rat islets. (**a**) Insulin content (left panel) and insulin release (right panel) after 45 min exposure in the presence or absence of 10 mM leucine (*n*=3–4). (**b**) Insulin content (left panel) and insulin secretion (right panel) after 45 min exposure to 2 or 20 mM glucose (*n*=3–4). (**c**) β-cell proliferation quantified by determining the percentage of insulin+ (ins+) and Ki67+ cells (*n*=3). (**d**) β-cell death assessed by scoring the percentage of insulin+ (ins+) cells labelled with TUNEL (*n*=3). All data are presented as means±s.d. as determined by ANOVA. *P<0.05, **P<0.01, ***P<0.001, ****P<0.001 (**a**–**d**). (**e,f**) Selected KEGG pathways upregulated (**e**) and downregulated (**f**) in adult rat islets. The bars show the Enrichment score (−log10 (*P*-value)) of the pathways in our data set. Modified Fisher's exact test (EASE cutoff *P*<0.05) was used to estimate the false discovery rate (FDR) and the thresholds to select significantly enriched KEGG pathways. (**g**) Scatter plot shows differentially expressed miRNAs between newborn and adult rat islets (*P*<0.05). Selected miRNAs up- and downregulated in adults are highlighted (*n*=4).

Pancreatic β-cells achieve a fully differentiated phenotype only after completion of a postnatal maturation process. The cellular composition of newborn rat islets is similar to that of adult animals with a slightly larger α-cell mass (Supplementary Fig. 1a,b). Ten-day-old rat β-cells display insulin content and basal insulin secretion comparable to those of adult β-cells (Fig. 1a,b). In addition, newborn and adult β-cells show a similar secretory response to 10 mM leucine (Fig. 1a). However, glucose-induced insulin secretion, an exclusive property of mature β-cells, is lacking in newborn β-cells (Fig. 1b) and these cells exhibit a replicative capacity five times higher than adult β-cells (Fig. 1c). In contrast, the apoptotic rate is not significantly different (Fig. 1d).

Analysis of islet gene expression by microarray revealed that postnatal maturation is associated with the upregulation of 2,001 and the downregulation of 1,691 mRNAs (fold change ≥2.0, *P*-value adjusted for multiple comparisons ≤0.05). The transcriptomic modifications are fully consistent with the phenotypic changes occurring during newborn β-cell maturation. Indeed, pathway analysis of the induced genes revealed enrichment of genes participating in insulin secretion and in the development of Type 2 diabetes (Fig. 1e). In contrast, the downregulated genes were preferentially involved in cell cycle, DNA replication and in amino acid and fatty acid metabolism (Fig. 1f). Using the same criteria of fold change and adjusted *P*-values, together with specification of a minimum abundance level for confident mRNA detection (see Methods), high-throughput RNA-sequencing of an independent set of islets revealed upregulation of 353 and downregulation of 575 mRNAs. Pathway analysis reached similar conclusions as for the microarray data. Quantitative PCR (qPCR) analysis of selected mRNAs, using a further independent set of islets, validated the findings of the array and sequencing platforms. All RNAs considered further in these studies exhibited differential expression in at least two of these three assays. Overall, integration of the gene expression changes identified by distinct methodologies revealed modifications in the expression of components of the same or of closely related pathways, including the enrichment of key genes involved in glycolysis (Supplementary Figs 2–5 and Supplementary Tables 1–4).

To determine whether miRNAs might contribute to the switch in gene expression programs, we compared the level of these small non-coding RNAs in islets of newborn (10-day-old) and adult rats (Fig. 1g). We detected 311 miRNAs and found that 68 of them displayed expression changes larger than twofold during β-cell maturation. Of these, 30 were upregulated and 38 downregulated in adult islets (Supplementary Table 5). As for mRNAs, similar results were obtained when an independent set of newborn and adult islet samples were analysed by small RNA-sequencing. The differences in expression of many of these miRNAs were confirmed by qPCR, both in rat and mouse islets (Table 1). Moreover, real-time PCR measurements in highly purified β-cells obtained by fluorescence-activated cell sorting (FACS) confirmed that the changes of most of these miRNAs occur in insulin-secreting cells (Supplementary Fig. 6). We found that several of the differentially expressed miRNAs belong to clusters of miRNAs that are generated from adjacent genomic loci, potentially from polycistronic precursors, or because of sequence similarity are classified into miRNA ‘families' (Supplementary Figs 7,8). Of note, members of the miR-17/92 and miR-106b/25 clusters as well as miR-215/194 and miR-192/194 clusters share the same seed sequence and are thus also classified as ‘families' likely to have overlapping targets (Supplementary Figs 7,8). These groups of miRNAs are therefore ideally suited to coordinate the modifications in gene expression programs taking place during postnatal β-cell maturation. Based on these observations, we selected for further analysis the miRNAs belonging to these clusters as well as a further group of individual miRNAs showing dramatic changes between newborn and adult islets (Table 1).

### Weaning drives microRNA changes and β-cell maturation

**Figure 2:**
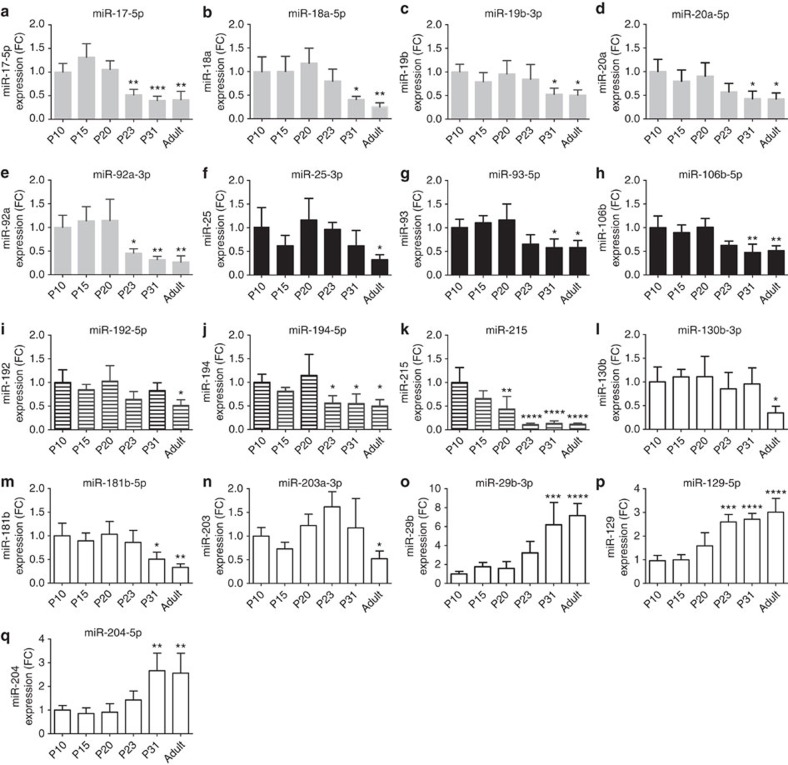
Weaning-dependent islet miRNA changes. qRT-PCR were performed using islet RNAs of rats from the indicated ages. miRNA levels were normalized to U6 and expressed in fold change (*n*=4–6 per group). The bars of miRNAs belonging to the same cluster are highlighted with the same shading. The bars of miRNAs that are not members of clusters are in white. Data are means±s.d. Statistical difference from P10 miRNA levels was assessed by one-way ANOVA with a Dunnett *post-hoc* test: *P<0.05, **P<0.01, ***P<0.001, ****P<0.0001.

We examined the kinetics of the modifications in miRNA expression by measuring levels of selected miRNAs at different time points during early postnatal life. We found that the majority of the modifications in miRNA expression occur between the 20th (P20) and the 31st (P31) postnatal day. In particular, the levels of miR-17-5p, miR-92a-3p, miR-194-5p and miR-129-5p are significantly modified at P23 (Fig. 2a,e,j,p) and altered levels of miR-18a-5p, miR-19b-3p, miR-20a-5p, miR-93-5p, miR-106b-5p, miR-181b-5p, miR-29b-3p and miR-204-5p reach significance at P31 (Fig. 2b–d,g,h,m,o,q). The expression of other miRNAs (miR-25-3p, miR-192-5p, miR-215, miR-130b-3p and miR-203a-3p) follow a different expression pattern (Fig. 2f,i,k,l,n). As expected, miRNAs belonging to the same genomic cluster displayed similar expression changes but, in some cases, with different kinetics. The modifications in miRNA profiles are specific to pancreatic islets and differ from those occurring during the same postnatal period in liver, muscle or brain (Supplementary Fig. 9).

**Figure 3:**
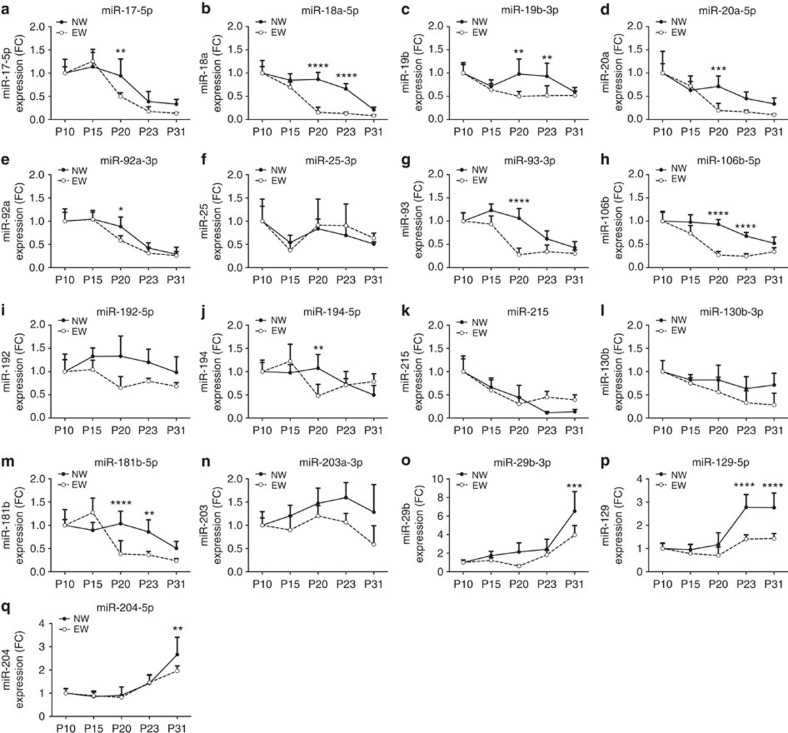
Prematurely weaned rats display earlier islet miRNA changes. qRT-PCR analysis was performed in samples from islets collected at the indicated postnatal days in rats weaned at P15 (EW, dotted line) or at P21 (NW, solid line) with chow diet. miRNA levels were normalized to U6 and expressed as fold change. Results correspond to means±s.d. (*n*=4–8 per group). *P<0.05, **P<0.01, ***P<0.001, ****P<0.0001 are determined by ANOVA comparing EW with NW at the indicated stage.

Our observations situate the critical period for the changes in islet miRNA expression near the time of weaning, which in rats takes place at P21. We reasoned that these processes may be driven by the drastic diet shift caused by the completion of a transition from maternal milk, rich in fat and proteins, to a carbohydrate-rich chow diet. To investigate this hypothesis, we weaned a group of rats at P15 and fed them with a starch-based diet. No significant weight loss was detected in prematurely weaned animals, ruling out the possibility of confounding effects linked to an eventual starvation of the pups (Supplementary Fig. 10). Remarkably, in animals subjected to early weaning (EW) with chow diet rich in carbohydrates the changes in the level of most miRNAs were accelerated compared to rats weaned at P21 (normal weaning: NW). Indeed, miR-17-5p, miR-18a-5p, miR-19b-3p, miR-20a-5p, miR-92a-3p, miR-93-5p, miR-106b-5p, miR-194-5p and miR-181b-5p are already reduced at P20 in EW pups (Fig. 3a–e,g,h,j,m); however, the upregulation of miR-29b-3p, miR-129-5p and miR-204-5p observed at the end of the postnatal period is lower in EW pups (Fig. 3o–q). Importantly, the expression pattern of miRNAs not observed to change near weaning time is not modified in animals prematurely withdrawn from the mother (Fig. 3f,i,k,l,n).

**Figure 4:**
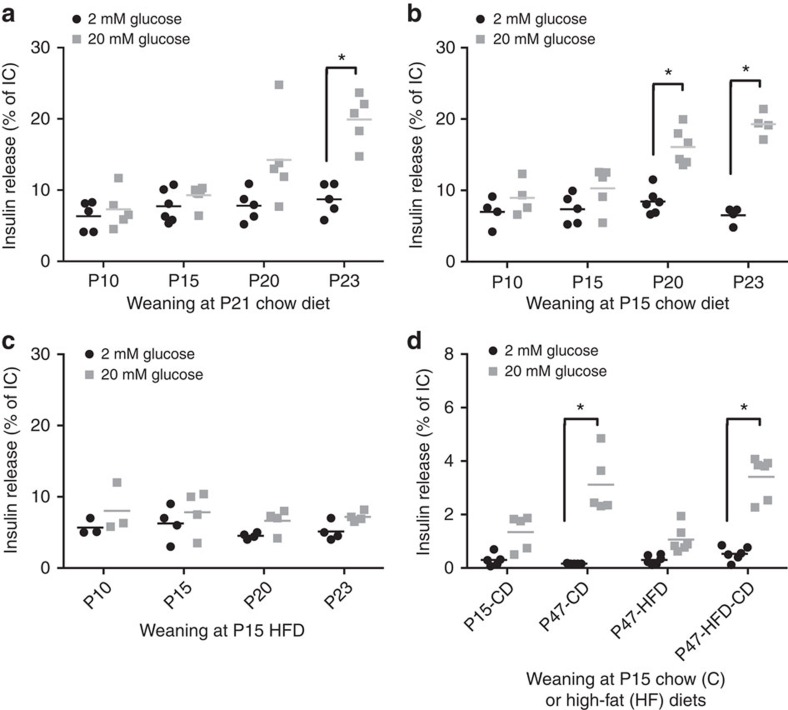
Impact of weaning time and diet composition on β-cell maturation. Insulin secretion in the presence of 2 or 20 mM glucose in islets isolated from rats of the indicated ages. (**a**) Data obtained from rats weaned at P21 on a carbohydrate-rich chow diet, (**b**) rats weaned at P15 on a carbohydrate-rich diet and (**c**) rats weaned at P15 on a high-fat diet (HFD). (**d**) Rats were weaned at P15 on carbohydrates- (CD) or fat-rich diet (HFD); a group of rats was weaned and kept on a HFD until P47 while a second group weaned on HFD was switched on a chow diet starting from P31 (HFD-CD). The amount of insulin secreted at 2 mM and 20 mM glucose was analysed at each time point. The results are means±s.d. (*n*=3–6 per group). *P<0.05 is determined by ANOVA followed by a Dunnett *post-hoc* test.

**Figure 5:**
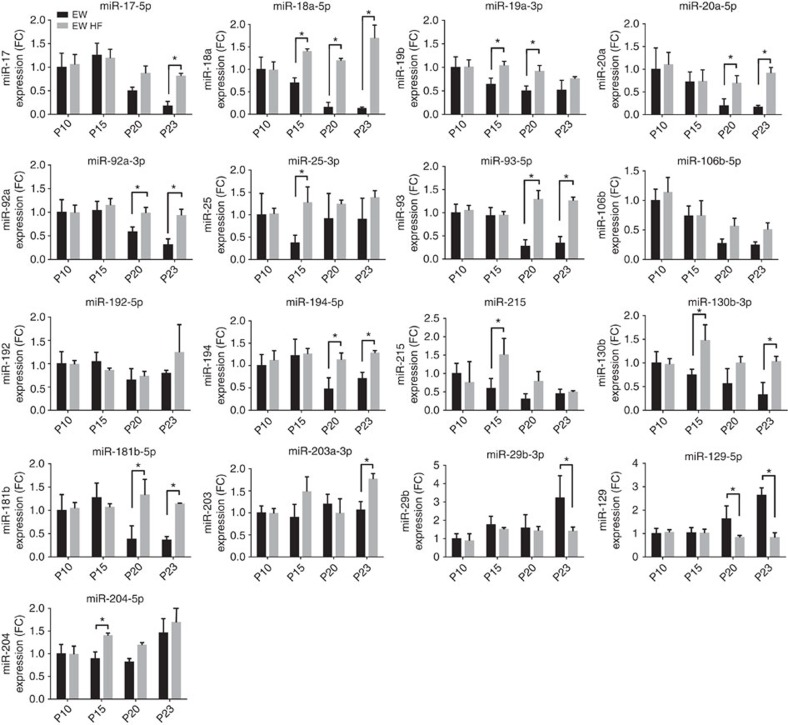
Impact of diet composition on miRNA expression changes. qRT-PCR were performed using islet RNAs of rats from the indicated ages in rats weaned at P15 with a carbohydrate-rich chow diet (black bars) or with a high-fat diet (grey bars). miRNA levels were normalized to U6 and expressed in fold change. Data are means±s.d. Statistical differences were assessed by one-way ANOVA with a Dunnett *post-hoc* test: *P<0.05.

To assess whether the changes in miRNA expression correlate with the acquisition of a mature secretory phenotype, we measured insulin release from the islets of the same animals. We found that in rats weaned normally at P21 the capacity to secrete insulin in response to 20 mM glucose coincides with the changes in miRNA expression and is fully achieved at P23 (Fig. 4a). In contrast, insulin content, basal secretion and leucine-stimulated insulin secretion remained constant from P10 to adults (Supplementary Fig. 11a,b). Moreover, changes in miR-17-5p, miR-106b-5p and miR-129-5p were correlated with the acquisition of glucose responsiveness during the postnatal period (Supplementary Fig. 12). Interestingly, premature weaning accelerated the acquisition of glucose-induced insulin secretion, with the typical response of P23 islets in NW observed as early as P20 (Fig. 4b). Again, basal insulin secretion, leucine-stimulated insulin secretion and insulin content were not affected by EW (Supplementary Fig. 11c,d) but the inverse correlation of miR-17-5p and miR-106b-5p with glucose responsiveness was even stronger in EW (Supplementary Fig. 12) and miR-181b-5p, which did not significantly decrease from P15 to P23 in NW, displayed a significant negative correlation to glucose responsiveness in EW. Taken together, our observations suggest that both islet miRNA changes and the acquisition of the capacity to secrete insulin in response to glucose are driven by the dietary shift associated with weaning. Analysis of β-cell replication at the same time points revealed a progressive decline from P10 to P20. The proliferation rate then remained stable until P31 without reaching the very low level observed in adult β-cells (Supplementary Fig. 13). The kinetics of altered β-cell proliferation were not modified in prematurely weaned animals. To assess whether the shift in diet composition is directly affecting the acquisition of glucose-induced insulin secretion, a group of newborn rats was weaned at P15 but was maintained on a high-fat diet (HFD). We found that in these animals the typical acquisition of glucose-induced insulin secretion at P23 was prevented (Fig. 4c). The effect of the diet persisted at least until P47 but was reversed if the animals were switched on a chow diet (Fig. 4d). Indeed, a group of rats initially weaned with a HFD but fed with a chow diet from P31 was able to release insulin in response to glucose as well as age-matched animals constantly maintained on a carbohydrate-rich diet (Fig. 4d). Moreover, the changes in islet miRNA levels normally occurring upon a shift to a carbohydrate-rich diet were prevented in animals prematurely weaned on a HFD (Fig. 5). Taken together, these data demonstrate that the shift from a fat-enriched diet to carbohydrate-enriched diet occurring during weaning plays an important role in the capacity of islets to release appropriate insulin in response to nutrient supply.

### miRNA changes initiate postnatal β-cell maturation

**Figure 6:**
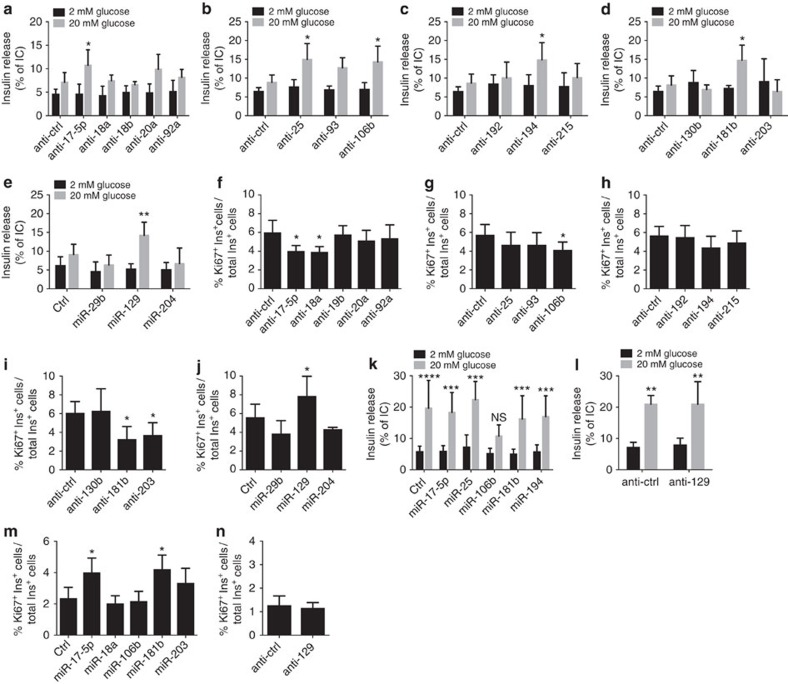
miRNAs contribute to the regulation of glucose-induced insulin secretion and β-cell proliferation. Dissociated P10 (**a**–**j**) or adult (**k**–**n**) rat islet cells were transfected with the indicated anti-miRs (**a**–**d**,**f**–**l**,**n**) or miRNA mimics (**e**,**j**,**k**,**m**). Two days later, insulin release and content was assessed in P10 (**a**–**e**) or adult (**k**,**l**) islet cells incubated at 2 or 20 mM glucose (*n*=5–7). The proliferating insulin+ cells from P10 (**f**,**g**,**h**,**i**,**j**) or adult (**m**,**n**) rats was assessed by Ki67 staining (*n*=6–7). All data are means±s.d. Statistical significance versus anti-ctrl or Ctrl was assessed by ANOVA: *P<0.05, **P<0.01, ***P<0.001, ****P<0.0001.

We next analysed the functional role of the miRNAs displaying expression modifications in the postnatal life and studied their involvement in β-cell maturation. For this purpose, we mimicked the changes in miRNA expression by transfecting 10-day-old rat islet cells with oligonucleotides that either block (anti-miRs) or increase (miRNA mimics) the level of the miRNAs (Supplementary Fig. 14a,b). We first assessed whether the modifications in miRNA expression affect insulin biosynthesis and secretion. Manipulation of selected miRNAs did not modify insulin content (Supplementary Fig. 15a-e), basal secretion (Fig. 6a–e) or leucine-stimulated insulin release (Supplementary Fig. 16a–e). However, when the postnatal maturation process was mimicked by reducing the level of miR-17-5p, miR-25-3p, miR-106b-5p, miR-194-5p, miR-181b-5p or by increasing the level of miR-129-5p (previously shown in Fig. 2), newborn β-cells acquired the capacity to release insulin in response to glucose (Fig. 6a–e). In contrast, changes in levels of the other miRNAs in these families were not sufficient to promote glucose responsiveness in newborn β-cells (Fig. 6a–e). Beside favouring the acquisition of a mature secretory phenotype, the reduction of miR-17-5p, miR-106b-5p and miR-181b-5p also resulted in decreased proliferation of newborn β-cells; reductions of miR-18a-5p and miR-203a-3p did not affect secretion but decreased proliferation (Fig. 6f,g,i). The other miRNAs had no impact on newborn β-cell replication (Fig. 6f-j) except for the increase of miR-129-5p that increased it further (Fig. 6j). The effects of individual miRNAs on glucose-induced insulin secretion and β-cell proliferation were not further amplified when all the miRNAs belonging to miR-17/92, miR-106b/25, miR-215/194 or miR-192/194 clusters were simultaneously reduced (Supplementary Fig. 17), suggesting that we had identified the most functionally important members of these clusters. Altogether, these data suggest that changes in the miRNA profile are instrumental in driving the acquisition of the mature β-cell phenotype. We then assessed whether fully functional adult β-cells could be reversed to an immature phenotype by modulating the same miRNAs. For this purpose, adult β-cells were transfected with miRNA mimics or anti-miRs to restore miRNAs to levels observed in newborn rats (Supplementary Fig. 14c,d). We found that except for the overexpression of miR-106b-5p, which led to a partial loss of glucose-induced insulin secretion (Fig. 6k), restoration of newborn levels of the other miRNAs did not have an impact on glucose-stimulated insulin release (Fig. 6k,l). Insulin content (Supplementary Fig. 15f,g) and insulin secretion under basal conditions (Fig. 6k,l) or in response to leucine (Supplementary Fig. 16f,g) were not altered. Overexpression of miR-17-5p and miR-181b-5p resulted in a 2-fold increase in the number of proliferating adult insulin-positive cells (Fig. 6m) whereas modifications in the level of the other miRNAs did not impact on adult β-cell replication (Fig. 6m,n). These findings suggest that the changes in miRNA expression observed in early postnatal life can drive the final differentiation of β-cells only in the particular gene expression context encountered in newborn β-cells.

### miRNAs drive expression of key genes for β-cell maturation

**Figure 7:**
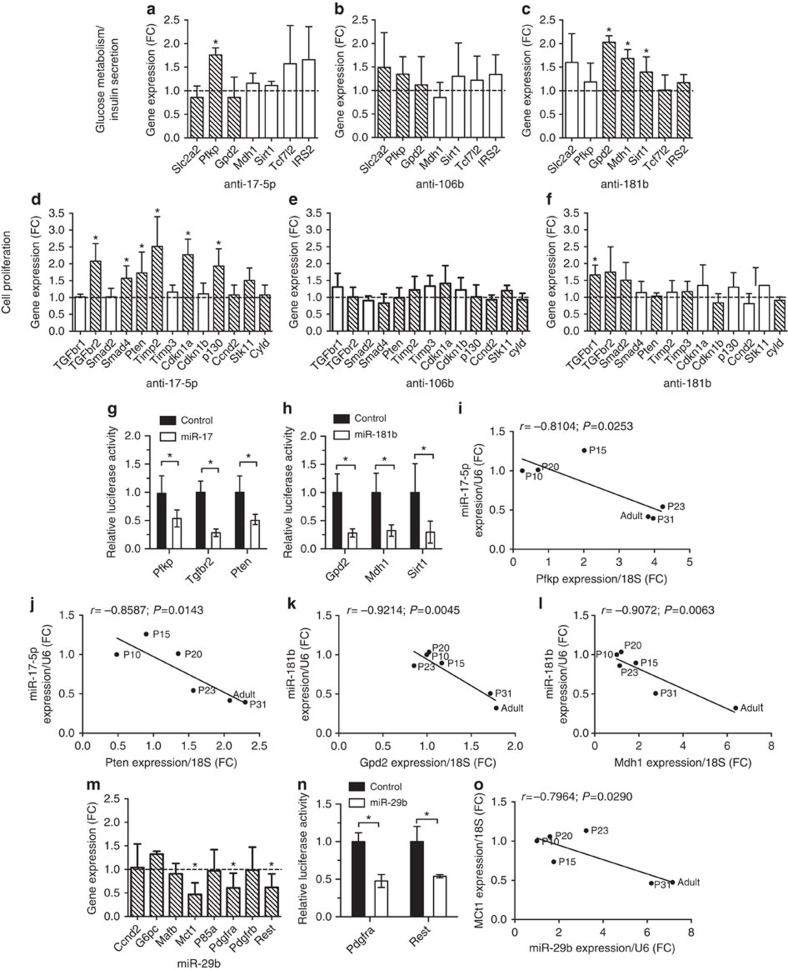
miR-17-5p and miR-181b orchestrate the expression of key β-cell genes. P10 islet cells were transfected with antimiR-17-5p (**a**,**d**), antimiR-106b (**b**,**e**), antimiR-181b (**c**,**f**) or miR-29b (**m**) (*n*=3–5). Genes involved in glucose metabolism, insulin secretion and proliferation were selected from the predicted targets of these miRNAs. The hatched bars highlight the predicted targets for the corresponding miRNAs. mRNA and miRNA levels were measured by qRT-PCR and are expressed as fold changes (FC) (*n*=4). Direct interaction with putative target mRNAs of miR17-5p (**g**), miR-181b (**h**) and miR-29b (**n**) was validated by luciferase reporter assays in the rat insulin-secreting cell line INS832/13. Vectors containing the 3'UTR of Pfkp, Tgfbr2, Pten, Gpd2, Mdh1, Sirt1, Pdgfra and Rest were co-transfected with either a control oligonucleotide or oligonucleotides mimicking the miRNAs of interest. The graphs show the correlations between the mRNA level of the identified target genes and miR-17-5-p (**i,j**), miR-181b (**k,l**), or miR-29b (**o**). Data are means±s.d. (*n*=3) and statistical significance were calculated by Student's *t*-test: *P<0.05. Correlations were established with Pearson correlation test.

To determine the mechanisms through which the differentially expressed islet miRNAs contribute to postnatal β-cell maturation, we delineated changes in gene expression specific to manipulation of individual miRNAs. In view of the functional effects observed when miR-17-5p, miR-106b and miR-181b are reduced in newborn islet cells on both, glucose responsiveness and cell proliferation, we examined whether relevant target genes controlling metabolism and cell replication predicted by miRSystem^32^ and miRWalk^33^ algorithms were upregulated upon their inhibition (Supplementary Table 6). We found that blockade of miR-17-5p resulted in the upregulation of phosphofructokinase (*Pfkp*), transforming growth factor β receptor 2 (*Tgfbr2*), *Smad4*, phosphatase and tensin homolog (*Pten*), TIMP metallopeptidase inhibitor 2 (*Timp2*), cyclin-dependent kinase inhibitor 1A (*p21; Cdk1a*) and retinoblastoma-like 2 (*p130; Rbl2*) (Fig. 7a,d). Despite sharing the same seed sequence, miR-106b-5p did not trigger the same expression changes as miR-17-5p and none of the genes tested was significantly modified upon its blockade (Fig. 7b,e). To test whether these genes are direct targets of miR-17-5p, luciferase constructs containing their 3′UTR sequences were co-transfected in the insulin-secreting cell line INS832/13 with a miR-17-5p mimic. We found that the expression of *Pfkp*, *Tgfbr2* and *Pten* constructs was indeed reduced by 50–70% upon miR-17-5p overexpression (Fig. 7g). Inhibition of miR-181b-5p resulted in increased expression of glycerol-3-phosphate dehydrogenase 2 (*Gpd2*), malate dehydrogenase 1 (*Mdh1*), sirtuin 1 (*Sirt1*) and transforming growth factor β receptor 1 (*Tgfbr1*) (Fig. 7c,f). Using a luciferase reporter assay, we indeed confirmed that *Gpd2*, *Mdh1* and *Sirt1* are direct targets of miR-181b (Fig. 7h). Consistent with the previous findings, we found that the levels of miR-17-5p and its targets *Pfkp* and *Pten* (Fig. 7i,j) and the levels of miR-181b-5p and those of *Gpd2* and *Mdh1* (Fig. 7k,l) are inversely correlated throughout the postnatal maturation process. No significant correlation was found for the other predicted mRNA targets of miR-17-5p or miR-181b-5p. Thus, downregulation of miR-17-5p and miR-181b-5p allow the expression of key metabolic enzymes and cell cycle regulators, reproducing the events occurring during postnatal β-cell maturation.

The most substantially upregulated miRNA during postnatal maturation was miR-29b-3p (Fig. 2o), although its overexpression did not affect glucose-induced insulin secretion (GSIS) acquisition or inhibit β-cell proliferation in the same manner as upregulated miR-129-5p (Fig. 6). Nonetheless, we observed a downregulation of the level of several predicted targets for miR-29b-3p (Supplementary Table 7). Indeed, when we reproduced the overexpression of miR-29b-3p occurring during postnatal β-cell maturation in 10-day-old rat islets we observed a striking reduction in the expression of the transcription factor *Rest*, and of *Mct1* and *Pdgfra* (Fig. 7m). Consistently, overexpression of miR-29b inhibited by about 50% the expression of luciferase constructs carrying the 3'UTR of *Pdgfra* and *Rest*, confirming a direct interaction with the miRNA (Fig. 7n). The increase of miR-29b-3p in islets throughout the postnatal period, was also negatively correlated with the repression of *Mct1*, a previously identified direct target of miR-29b^34^ (Fig. 7o). Low expression of Mct1 is a key feature of mature β-cells, which prevents pyruvate entry into the TCA cycle from lactate conversion and is thought to prevent consequent ‘short-circuiting' of glucose-dependent ATP production that triggers GSIS. Taken together, these experiments provide an initial explanation for the molecular mechanisms causing the phenotypic modifications occurring during the maturation of β-cells.

### Downstream signalling elicited by miR-17-5p modification

**Figure 8:**
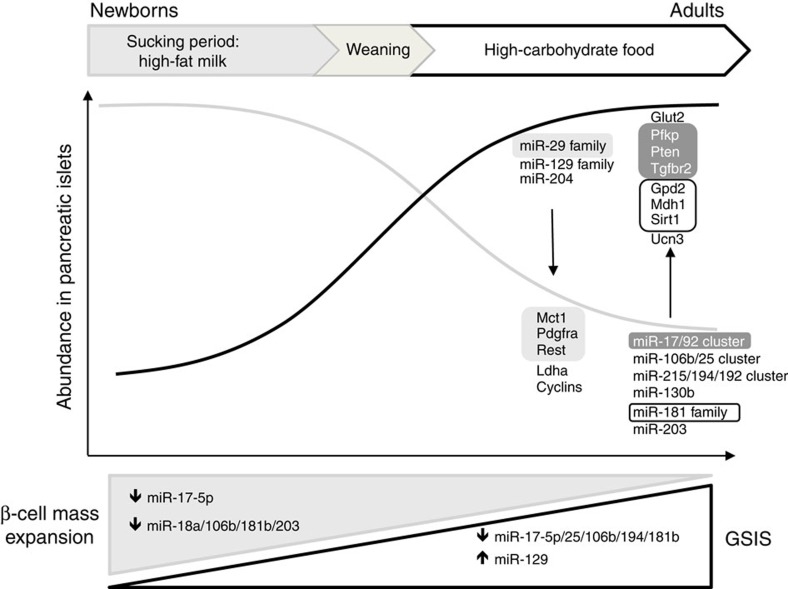
Schematic representation of transcriptomic and phenotypic changes occurring during postnatal β-cell maturation. Weaning coincides with major modifications of microRNA and mRNA expression in pancreatic islets. The decrease of miR-17-5p and miR-181b-5p and the concomitant increase of miR-29b-3p are inversely correlated with expression changes of directly and indirectly targeted genes essential to achieve full β-cell maturation (highlighted). These modifications allow the acquisition of glucose-induced insulin secretion (GSIS) and reduce β-cell proliferation to define the size of the adult β-cell mass.

We focused on miR-17-5p that appeared to control a large number of mRNA expression modifications, and overexpressed it in P10 islet cells (Supplementary Fig. 18). To empirically identify direct mRNA targets, we used the unbiased approach of Ago2 immunoprecipitation and subsequent RNA-sequencing of both the Ago2 and global mRNA fractions^35^. Firstly, at the global mRNA level, miR-17-5p overexpression altered the abundance of 309 mRNAs (false discovery rate ≤0.1) that had also passed detection criteria in the prior comparisons of adult and P10 islets. 185 of these mRNAs (60%) were altered in the opposite direction in adult *vs* P10 islets (false discovery rate≤0.1), in which miR-17-5p expression is decreased, and a further 64 mRNAs (21%) were altered in the opposite direction in adult *vs* P10 islets but did not reach statistical significance (Supplementary Data 1). Bioinformatic analysis confirmed the enrichment of genes involved in cell proliferation, TGFβ signalling pathway and cell growth as previously observed by quatitative PCR with reverse transcription (qRT-PCR) (Supplementary Data 2). Thus, a change in miR-17-5p alone was able to recapitulate a substantial fraction of the differences in gene expression observed between adult and P10 islets. Secondly, to identify direct mRNA targets of overexpressed miR-17-5p, we searched for mRNAs whose abundance increased in the Ago2 immunoprecipitate (RISC fraction), with either a parallel decrease in the global mRNA fraction or no significant change in global mRNA (indicating RISC-mediated transcript degradation or translational suppression, respectively). One hundred thirteen mRNAs were thus defined as putative direct targets of miR-17-5p in P10 islets (Supplementary Data 3). Bioinformatic analysis showed an increased RISC incorporation of transcripts involved in cell cycle regulation (Supplementary Table 8), similarly to those mRNAs altered without direct RISC involvement (Supplementary Data 1). Taken together, these data show that we have identified direct, translationally suppressed mRNA targets of miR-17-5p through RISC association experiments, together with a number of putative direct targets of miR-17-5p using target prediction algorithms, miRNA inhibition and mRNA measurements. Overall, regardless of direct or indirect control of these mRNAs by miRNAs, we have identified critical mRNAs that respond to miRNA reprogramming and that likely contribute to the shift in gene expression programs needed for postnatal β-cell maturation and acquisition of the distinct functional identity of adult islets (Fig. 8).

## Discussion

MiRNAs are important players in the onset of chronic metabolic diseases such as diabetes. These non-coding RNAs are central actors in the embryonic differentiation of insulin-producing cells and in the control of mature β-cell activities^25^,^36^ but, so far, no information has been available about their possible contribution to postnatal β-cell maturation, a critical process for the acquisition of adult β-cell function. A better understanding of the mechanism driving this essential process will be instrumental for the design of cell-based strategies for the treatment of diabetes.

In agreement with previous reports^8^,^9^,^37^,^38^, we detected marked phenotypic differences between 10-day-old and adult rat β-cells; in particular, an absent secretory response to increased glucose levels and higher proliferation rates in newborn β-cells. A recent study carried out in islets from infants suggests that analogous functional differences may exist in humans^39^. We found that the phenotypic characteristics of newborn β-cells are linked to a very low expression of genes involved in insulin secretion and glucose metabolism and to a marked expression of genes promoting proliferation and the metabolism of amino acids and fatty acids. The data obtained in our global transcriptomic survey corroborate those of previous studies focusing on specific metabolic genes and developmentally regulated transcription factors^10^,^11^,^13^,^40^,^41^,^42^.

Systematic analysis of the miRNA profiles of newborn and adult β-cells revealed major differences in the level of these non-coding RNAs and highlighted selective changes of several clusters and families of miRNAs. We observed that most of these changes occur between the 20th and 31st postnatal day and correlate with the acquisition of β-cell glucose responsiveness. This critical time window corresponds to the weaning period when nutritional input is drastically modified by the replacement of high-fat maternal milk with a carbohydrate-rich diet. Our findings are in agreement with studies that situate the acquisition of mature β-cell functionality around weaning time^9^,^24^ and that postulate a key role for weaning in physiologic adaptations^19^. Interestingly, many of the relevant changes in miRNA expression and the acquisition of a mature secretory phenotype take place earlier in newborn rats that are fed a carbohydrate-rich diet (prematurely weaned) starting from day 15 rather than day 21. These observations are consistent with a study demonstrating induction of key islet genes involved in glucose metabolism, increased insulin expression and hyperinsulinemia in 4-day-old rat pups fed with a high-carbohydrate milk formula^22^. The accelerated acquisition of these phenotypic traits is likely to be part of a metabolic adaptation that permits maintenance of normoglycemia in the context of a carbohydrate-rich diet via increased insulin secretion. In contrast, weaning the rats on a HFD prevented most miRNA changes and the acquisition of glucose-induced insulin secretion, suggesting that these events are driven by the exposure to a carbohydrate-rich diet and the consequent increase in the insulin needs rather than to a particular developmental stage. The dietary shift elicits metabolic adaptations that are likely to extend far beyond changes in β-cell properties alone. Indeed, after weaning increased hepatic expression of glycolytic and lipogenic enzymes consequent to a rise in plasma insulin levels have been reported^19^,^20^,^43^,^44^. While speculative, some of these adaptive gene expression changes may possibly be driven by modifications in the level of liver miRNAs detected in our study (Supplementary Fig. 9).

The robust proliferative capacity of newborn β-cells was found to decline between the 10th and the 20th postnatal day but, consistent with previous studies in rodents showing continuous β-cell mass expansion over the first month of life^45^,^46^, the replicative rate at P31 was still higher than that observed in adults and was not affected by premature weaning. Very recently, Dor and colleagues reported that premature weaning of mice on a HFD does not affect basal β-cell expansion but impairs their capacity to proliferate in response to glucose^24^. These findings are fully consistent with those presented here. In fact, this compensatory, glucose-responsive β-cell mass expansion is likely to rely on the same glucose sensing mechanisms controlling insulin secretion that are acquired upon weaning. In view of these observations, it is tempting to speculate that some of the miRNAs characterized in the present study may thus contribute to compensatory β-cell mass expansion in response to a glucose challenge.

To establish a causal link between the modifications in the miRNA profile and the postnatal maturation process, we experimentally reproduced in newborn islet cells most of the relevant miRNA changes and scrutinized their impact on insulin secretion and cell proliferation. Indeed, mimicking the changes in the level of several miRNAs altered at weaning (miR-17-5p, miR-25-3p, miR-106b-5p, miR-194-5p, miR-181b-5p and miR-129-5p) established a premature glucose-induced secretory response in newborn β-cells. Moreover, manipulating the level of some miRNAs (miR-17-5p, miR-18a-5p, miR-106b-5p, miR-181b-5p and miR-203a-3p) reduced the proliferation rate of immature β-cells, suggesting that the presence of low amounts of these miRNAs in adult β-cells contributes to their limited proliferative capacity. These data are consistent with the roles attributed to several of these miRNAs in studies carried out in mature β-cells. Indeed, reduction of miR-25-3p was shown to enhance glucose-induced insulin secretion in adult β-cells^47^ and the clusters miR-17/92, miR-106b/25 and miR-181b-5p were postulated to control β-cell proliferation^48^,^49^. Remarkably, the level of most miRNAs identified as regulators of β-cell maturation is reduced, rather than increased, during postnatal development. This observation supports the fascinating hypothesis put forward by Rutter and Hornstein groups, suggesting that a specific set of miRNAs needs to be disallowed to achieve proper β-cell maturation^34^,^50^. The precise mechanisms underlying the changes in miRNA expression remain to be explored. The acquisition of a mature β-cell secretory phenotype was previously proposed to be driven by increased expression of the transcription factor Mafa^51^. Future studies will need to assess the existence of a possible link between the induction of Mafa or other transcription factors and switches in miRNA transcription.

In our study, we were able to show that the downregulation of specific miRNAs is instrumental for the activation of gene expression programs culminating in the acquisition of a fully mature β-cell phenotype. In fact, blockade of miR-17-5p and miR-181b-5p permitted the expression of several key limiting enzymes for glucose metabolism such as *Pfkp*, *Gpd2*, *Mdh1* and of genes regulating cell proliferation such as *Pten*, *Tgfbr1/2* and *Cdkn1a*. Interestingly, *Gpd2* and *Pfk* were both found to be expressed at lower levels in pancreatic islets of Type 2 diabetic subjects^52^,^53^ and were associated with impaired glucose metabolism and loss of β-cell function^54^,^55^. By identifying key mRNA regulators of metabolism and β-cell function as direct targets of miR-17-5p and miR-181b, our data raise the hypothesis that misregulation of miRNAs (and consequently the expression of their targets during postnatal life) may predispose individuals to metabolic disturbances and diabetes onset via interference with postnatal β-cell mass expansion. miR-29b-3p is among the most abundant miRNAs present in pancreatic islets^56^, which we also demonstrated in our miR-sequencing data set. The strong upregulation of miR-29b-3p occurring during the postnatal maturation process is likely to ensure proper silencing of key transcription factors such as *Rest*, which is absent in mature insulin-producing cells^57^, and of other factors such as *Mct1* and *Pdgfra* that belong to a group of genes specifically disallowed in adult, fully functional mouse islets^13^,^58^,^59^. Our findings demonstrating direct targeting of *Rest* and *Pdgfra* complement a previous report of miR-29b-3p binding to the 3'UTR of *Mct1* (ref. 34) and strengthen the hypothesis that this miRNA is an important factor in programming and maintaining β-cell identity during postnatal development, although its manipulation in our studies was not sufficient on its own to provoke alterations in GSIS acquisition or β-cell proliferation.

In conclusion, this study demonstrates that miRNAs are key regulators of postnatal β-cell maturation and essential players in the metabolic adaptation of newborns to changes in nutrient supply. Our observations suggest that malnutrition during early life, within a critical time window corresponding to the completion of endocrine pancreas maturation, may affect the metabolic reprogramming necessary to the acquisition of a fully mature β-cell phenotype and to the establishment of an appropriate β-cell mass. In adulthood, this could potentially have an impact on the capacity of β-cells to meet conditions of increased insulin demand such as pregnancy and obesity, predisposing the affected individuals to the development of gestational and Type 2 diabetes^18^,^60^. A better understanding of these critical events occurring early in life will help in elucidating the causes of individual diabetes susceptibility in the adult.

## Methods

### Animals

Sprague Dawley rats were obtained from Janvier Laboratories. Animal experimentation was carried out in Lausanne, Switzerland. All procedures were performed in accordance with the National Institutes of Health guidelines, and were approved by the ‘Service de la consommation et des affaires veterinaires', Epalinges, Switzerland (authorization VD2608).

### Weaning procedure

At birth, litters were adjusted to 10–12 offsprings per dam to standardize mother milk availability. Newborn pups (males and females) were nursed until they were killed. At P21 (normal weaning, NW), at P15 (EW) or at P31, the pups were weaned on a standard chow diet containing 18.5% crude proteins, 4.5% fat and 54% carbohydrates or on a HFD providing 5.24 Kcal g^−1^, 20% proteins, 60% fat, 20% carbohydrates. Food was crushed and put directly above bedding for 5 days to ensure food availability to the prematurely weaned rats.

### RNA assays

Total RNA was extracted with the miRNeasy kit (Qiagen) except for mRNA- and miRNA-sequencing for which Trizol was used. For mRNA-sequencing, each biological replicate was a pool of islets of five P10 pups or of a single adult rat. For microarray analysis and real-time PCR, pancreatic islets from two P10 pups were pooled while islets from P15, P20, P23, P31, P47 and adult rats were extracted individually. For qPCR measurements, islets from either five to seven pups or from two adult rats were pooled. miRNA profiling was performed at the Genomic Technologies Facility of the University of Lausanne using Agilent Technologies miRNA Gene Microarrays. mRNA profiling was performed by Arraystar using Agilent Technologies and data analysis was performed using Agilent GeneSpring GX v11.5.1 software. For mRNA-sequencing the RNA was converted into a library of template molecules for Illumina single-end sequencing as previously described^35^. miRNA-sequencing was performed from the same input RNA samples, using the Illumina TruSeq small RNA-sequencing kit as previously described^61^. miRNA- and mRNA-sequencing was performed at Washington University GTAC (indexed Illumina single-end, 50 nt sequencing on a HiSeq 2500 instrument) and analyses of raw read data performed as previously described^62^. miRNA abundances from sequencing analyses are presented as Reads per million reads mapped to miRNAs; mRNA abundances are presented as FPKM (Fragments Per Kb of exon per Million reads mapped to mRNAs). However, differential expression was calculated using raw read counts as input to DESeq (see **Statistics**). Biological function and pathway analyses were performed using DAVID^63^.

Mature miRNA and mRNA levels were assessed by qRT-PCR using the miScript II RT kit (Qiagen). miScript primer assays and primer sequences are provided in Supplementary Table 9. miRNA expression was normalized to the level of U6. mRNA expression was normalized to the amount of 18S rRNA.

### Cell culture

Pancreatic islets were isolated by collagenase (Roche) digestion^64^ and purified on a Histopaque (Sigma-Aldrich) density gradient. They were cultured in RPMI 1640 Glutamax medium (Invitrogen, Carlsbad, CA, USA) supplemented with 10% fetal calf serum (Amimed), penicillin 50 U ml^−1^, streptomycin 50 μg ml^−1^, Na Pyruvate 1 mM and 250 μmol l^−1^ Hepes.

### Cell transfection

Transfection of dissociated rat islet cells with miScript miRNA inhibitors (anti-miR) (Qiagen) or with oligonucleotide duplexes (Eurogentec) was performed using Lipofectamine 2000 (Invitrogen) as previously described^65^.

### Fluorescence-activated cell sorting (FACS)

Dissociated islet cells from newborn and adult rats were sorted by FACS based on β-cell autofluorescence, as previously described^66^. Purity of sorted β-cells was evaluated by immunocytochemistry using insulin (Dako: #A0564) and glucagon (Abcam: #ab10988) antibodies. Our FACS contained on average 94±1% β-cells and 3–4% of α-cells.

### Insulin secretion

Whole islets and dissociated rat islet cells were preincubated with KREBS buffer (127 mM NaCl, 4.7 mM KCl, 1 mM CaCl2, 1.2 mM KH2PO4, 1.2 mM MgSO4, 25 mM HEPES, 5 mM NaHCO3) containing 2 mM of glucose (basal condition) during 30 min at 37 °C. The medium was then replaced by either the same buffer (basal condition) or a KREBS buffer supplemented with 20 mM of glucose (stimulatory condition). After 45 min, the supernatants were collected and cellular insulin contents were recovered in EtOH acid (75% EtOH, 0.55% HCl). The amount of insulin in the samples was determined by ELISA (Mercodia).

### Immunocytochemistry

Dissociated rat islet cells were plated on poly-L-lysine-coated glass coverslips. The cells were fixed in cold methanol and incubated with phosphate-buffered saline supplemented with 0.5% saponin (Sigma-Aldrich) for 15 min. After 30 min incubation in blocking buffer (phosphate-buffered saline supplemented with 0.5% saponin and 1% bovine serum albumin), the cells were incubated for 1 h at room temperature with primary antibodies at the following dilutions: 1:1,300 rabbit anti-Ki67 (Abcam: #ab15580); 1:500 guinea pig anti-insulin (Abcam: #ab10988). The coverslips were then incubated during 1 h at room temperature with goat anti-rabbit Alexa-Fluor-488 and goat anti-guinea pig Alexa-Fluor-555 diluted at 1:400 (Invitrogen: # A11008 and # A21435. respectively). Before being mounted on microscope glass slides using FluorSave mounting medium (VWR International SA) the coverslips were treated for 10 min at room temperature with Hoechst 33342 (Invitrogen). The cells were visualized with a Zeiss Axiovision fluorescence microscope.

### Immunohistochemistry

Animals were killed and the pancreases fixed in 10% neutral buffered formalin (Sigma-Aldrich) for 24 h and embedded into paraffin for sectioning. The slices were stained using the following primary antibodies: 1:500 rabbit anti-Ki67 (Abcam: # ab15580), 1:200 guinea pig anti-insulin (Abcam: # ab7842), 1:200 rabbit anti-glucagon (Abcam: # ab10988). Goat anti-rabbit Alexa-Fluor-488, goat anti-guinea pig Alexa-Fluor-555 and goat anti-mouse Alexa-Fluor-555 diluted at 1:400 (Invitrogen: # A11008, # A21435 and # A21422, respectively) were used as secondary antibodies. TUNEL staining was performed using the In Situ Cell Death Detection Kit (Roche). Cell nuclei were visualized with Hoechst 33342. Images were collected using a Zeiss Axiovision fluorescence microscope.

### Luciferase assays

Luciferase reporter plasmids were generated by cloning about 200 nucleotides of the 3′UTR of rat Pfkp, Tgfbr2, Pten, Gpd2, Mdh1, Sirt1, Pdgfra and Rest, containing the putative binding sites of miR-17-5p, miR-181b or miR-29b. Luciferase activity was measured in INS832/13 cells with a dual-luciferase reporter assay (Promega, Madison, WI, USA) 3 days after transfection. Renilla luciferase activity was normalized for transfection efficiency to the SV40-driven Firefly activity generated from a co-transfected PGL3 promoter vector (Promega).

### Ago2 immunoprecipitation and RISC-sequencing

Ago2 immunoprecipitation and preparation and sequencing of RNA libraries from RISC fractions were performed as described^35^,^67^. Raw read analysis was carried out as described^62^. Biological function and pathway analyses were performed using DAVID^63^.

### Statistics

Values are expressed as mean±s.d. Statistical analysis was performed using one-way ANOVA with Dunnett's *post-hoc* test for multiple comparisons or two-way ANOVA with Tukey's *post-hoc* test for multiple comparisons. Student's t-test was used for unpaired comparisons. *P*-values were adjusted by the Benjamini–Hochberg method, controlling for false discovery rate for the miRNA microarray. Correlation coefficients and significance were derived from Pearson rank correlation test. Differential expression from sequencing reads were computed on read count data using the DESeq package in R (ref. 68) as previously described^62^. miRNAs with an abundance <10 RpM in more than 50% of samples were designated as ‘below read cutoff'. Similarly, mRNAs whose reads represented <1/100,000th of the total reads were filtered out, leaving ∼7,300 mRNAs for further analysis.

## Additional information

**Accession codes:** All miRNA and mRNA microarray and RNA-sequencing data have been deposited in NCBI GEO under accession code GSE61182.

**How to cite this article**: Jacovetti, C. *et al*. Postnatal β-cell maturation is associated with islet-specific microRNA changes induced by nutrient shifts at weaning. *Nat. Commun*. 6:8084 doi: 10.1038/ncomms9084 (2015).

## Supplementary Material

Supplementary Information Supplementary Figures 1-18 and Supplementary Tables 1-9

Supplementary Data 1 Effects of miR-17-5p overexpression on the mRNA transcriptome of P10 rat islet cells.

Supplementary Data 2 List of gene ontology functions significantly enriched amongst miRNA-independent mRNA changes upon miR-17-5p overexpression in 10-day-old rat islet cells.

Supplementary Data 3 Effects of miR-17-5p overexpression on RISC-targeted mRNAs in P10 islet cells.

## Figures and Tables

**Table 1:** Islet microRNA expression changes assessed by microarray and qRT-PCR between newborn and adult rat and mouse islets.

	Microarray from rat islets				qRT-PCR from rat islets		qRT-PCR from mouse islets	
microRNA	Fold change adult vs P10	Adjusted *P*-value (FDR)	Mean P10 Log2	Mean adults Log2	Fold change adult vs P10	*P*-value	Fold change adult vs P10	*P*-value
miR-17-5p	−4.2	2.28e−04	8.01	5.94	−3.5	1.93e−02	−3.5	2.62e−02
miR-18a	−3.7	1.97e−04	6.44	4.56	−3.5	4.92e−02	−2.6	1.71e−02
miR-19b	−4.5	5.98e−04	11.30	9.13	−3.6	3.79e−03	−3.8	5.90e−03
miR-20a	−3.6	3.65e−04	9.50	7.64	−4.1	1.89e−02	−2.8	3.28e−02
miR-92a	−4.3	4.47e−03	10.43	8.31	−7.3	3.41e−02	−6.3	2.14e−02
miR-25	−2.3	8.46e−03	10.00	8.81	−1.9	6.45e−04	−11.4	2.07e−02
miR-93	−2.9	2.67e−04	8.48	6.97	−2.3	3.60e−02	−4.1	1.33e−02
miR-106b	−2.2	5.11e−04	10.96	9.79	−3.2	2.02e−02	−10.1	2.01e−02
miR-192	−6.8	1.37e−02	10.85	8.09	−5.0	5.00e−02	−1.7	3.72e−03
miR-194	−9.5	8.70e−03	10.16	6.91	−4.2	2.54e−02	−1.3	2.68e−02
miR-215	−139.2	5.82e−04	9.62	2.50	−112.9	2.44e−03	−2.0	1.79e−01
miR-130b	−5.3	8.92e−04	7.96	5.57	−5.1	1.23e−02	−5.4	5.90e−03
miR-181b	−5.1	3.86e−03	7.68	5.35	−6.7	2.45e−03	−2.6	4.74e−02
miR-203	−6.9	2.58e−03	7.60	4.81	−6.4	8.85e−03	−7.6	2.14e−01
miR-29b	8.6	2.53e−04	10.60	13.71	11.6	4.72e−03	14.8	3.95e−02
miR-129	5.7	4.60e−03	5.86	8.36	3.4	1.35e−02	3.7	1.44e−02
miR-204	3.8	3.65e−04	7.51	9.44	4.5	3.96e−02	1.8	1.71e−01

FDR, false discovery rate; qRT-PCR, quatitative PCR with reverse transcription.

Relative expression of the 17 selected miRNAs as determined by microarray. The mean expression of each group is presented on a log2 scale and adjusted p-values are the smallest FDR for which the test can be called ‘significant'. Fold changes in miRNA expression as confirmed by qRT-PCR in 10-day-old *vs* adult rat islets (*n*=4) and in 10-day-old *vs* adult mouse islets (*n*=3) (*P*<0.05). For the microarray data, the *P*-values were adjusted using the Benjamini–Hochberg method to evaluate the FDR.
