# Supplementary material for: Postnatal β-cell maturation is associated with islet-specific microRNA changes induced by nutrient shifts at weaning
Source: Nat Commun. 2015 Sep 2;6:8084. doi: 10.1038/ncomms9084 (PMC4569696; doi:10.1038/ncomms9084)
Supplement: Supplementary Information — Supplementary Figures 1-18 and Supplementary Tables 1-9 [file ncomms9084-s1.pdf]

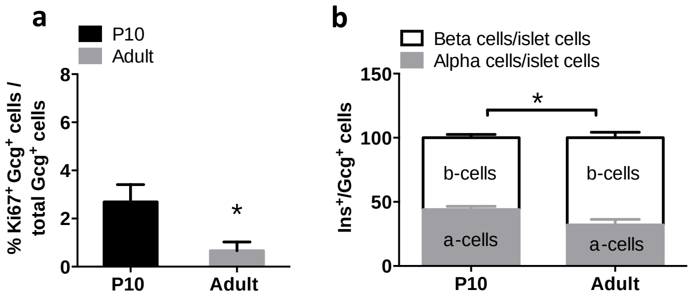

Supplementary Figure 1. Alpha-cell proliferation and mass in newborn and adult islets. (**a**) Proliferation was assessed by scoring the Ki67-positive ($Ki67^+$) cells co-labelled with glucagon (Gcg^+) (**b**) The fraction of alpha and β-cells in neonatal and adult islets was determined by immunohistochemical staining for glucagon and insulin, respectively. Data are means ± SD; n=3 per group; Student's t-test *P<0.05).

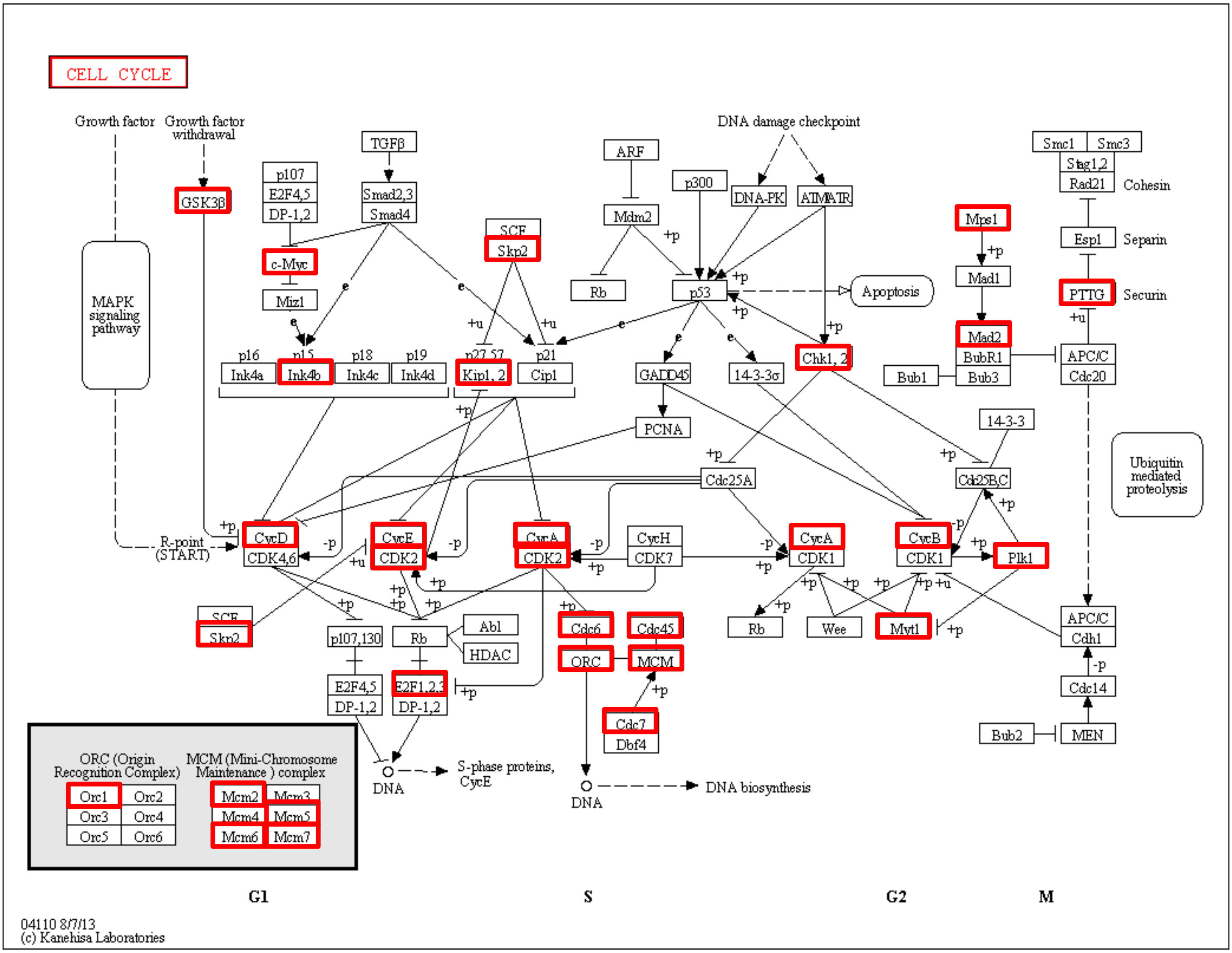

Supplementary Figure 2. Enriched cell cycle pathway in adult versus 10-day-old rat islets defined by KEGG pathway analysis. The genes down-regulated are boxed in red. Supplementary table 1 shows the fold change (FC) values reported from the array (n=3 per group) and confirmed by qRT-PCR (n=4 per group) and RNA-sequencing (n=3 per group). (a: absent; Adj-p value <0.05).

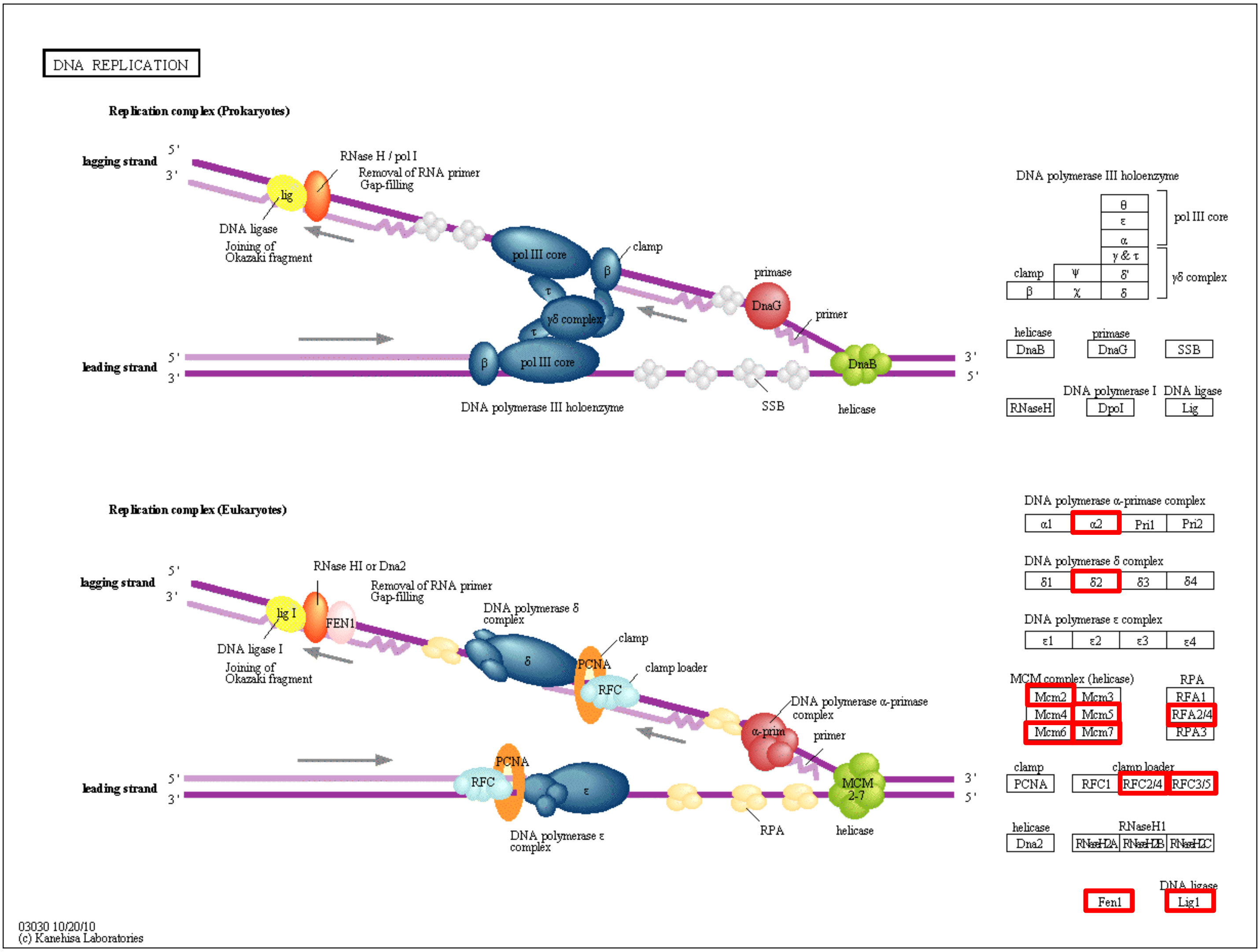

Supplementary Figure 3. Enriched DNA replication pathway in adult versus 10-day-old rat islets defined by KEGG pathway analysis. The genes down-regulated are boxed in red. Supplementary table 2 shows the fold change (FC) values reported from the array (n=3 per group) and confirmed by qRT-PCR (n=4 per group) and RNA-sequencing (n=3 per group). (a: absent; Adj-p value <0.05).

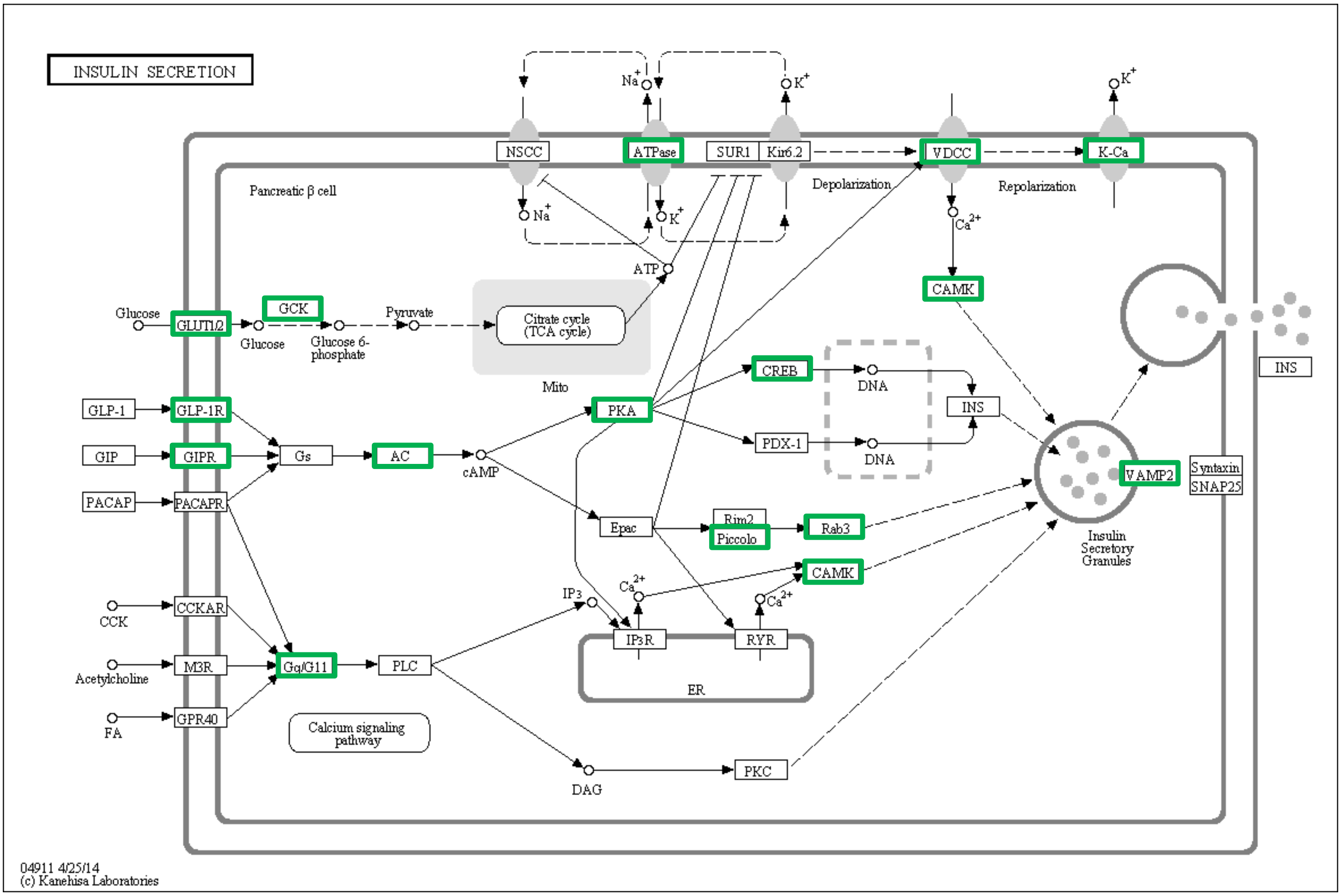

Supplementary Figure 4. Enriched insulin secretion pathway in adult versus 10-day-old rat islets defined by KEGG pathway analysis. The genes up-regulated are boxed in green. Supplementary table 3 shows the fold change (FC) values reported from the array (n=3 per group) and confirmed by qRT-PCR (n=4 per group) and RNA-sequencing (n=3 per group). (a: absent; Adj-p value <0.05).

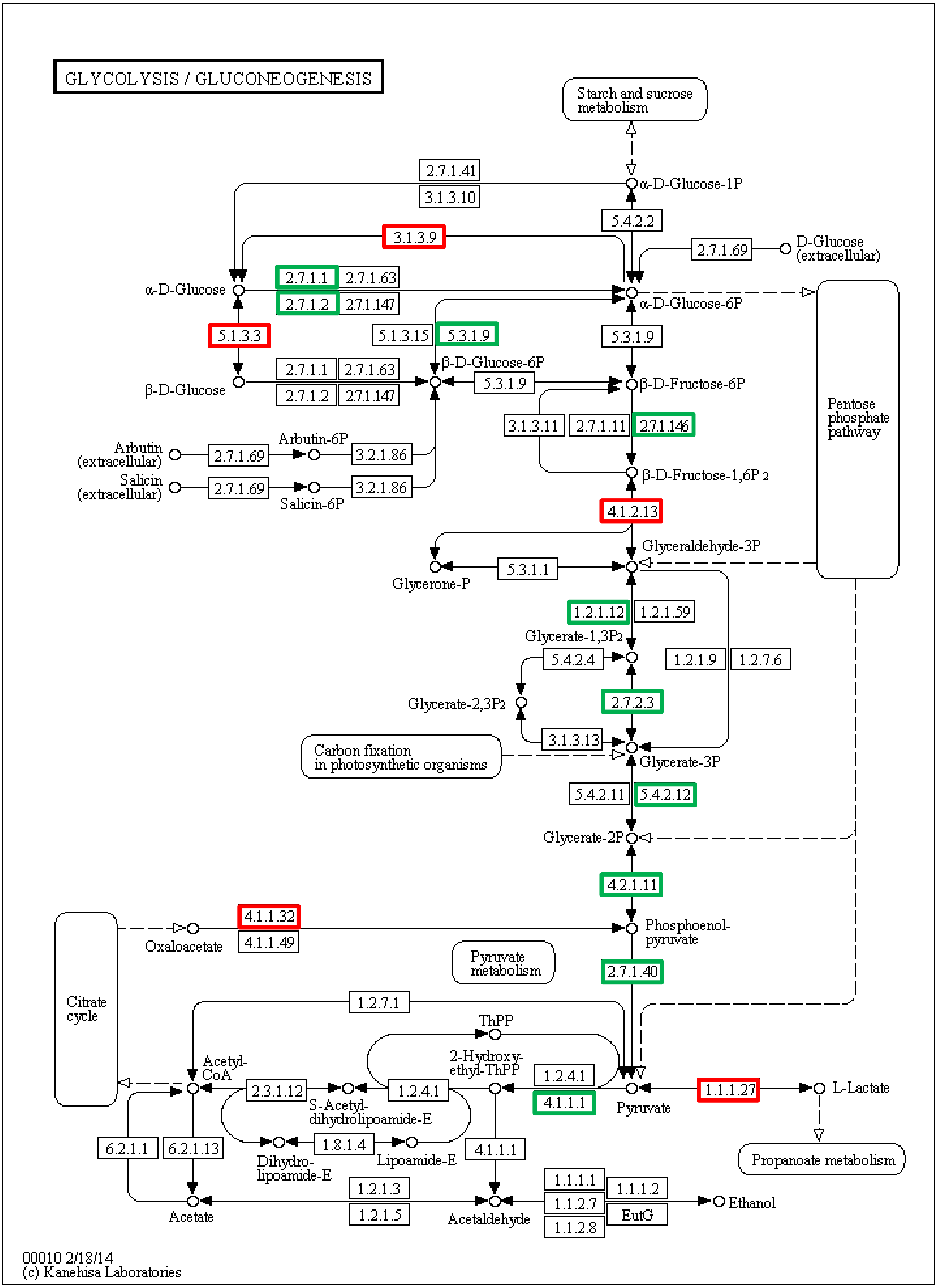

Supplementary Figure 5. Enriched glycolysis/gluconeogenesis pathway in adult versus 10-day-old rat islets defined by KEGG pathway analysis. The genes down-regulated are boxed in red and the genes up-regulated are boxed in green. Supplementary table 4 shows the fold change (FC) values reported from the array (n=3 per group) and confirmed by qRT-PCR (n=4 per group) and RNA-sequencing (n=3 per group). (a: absent; Adj-p value <0.05).

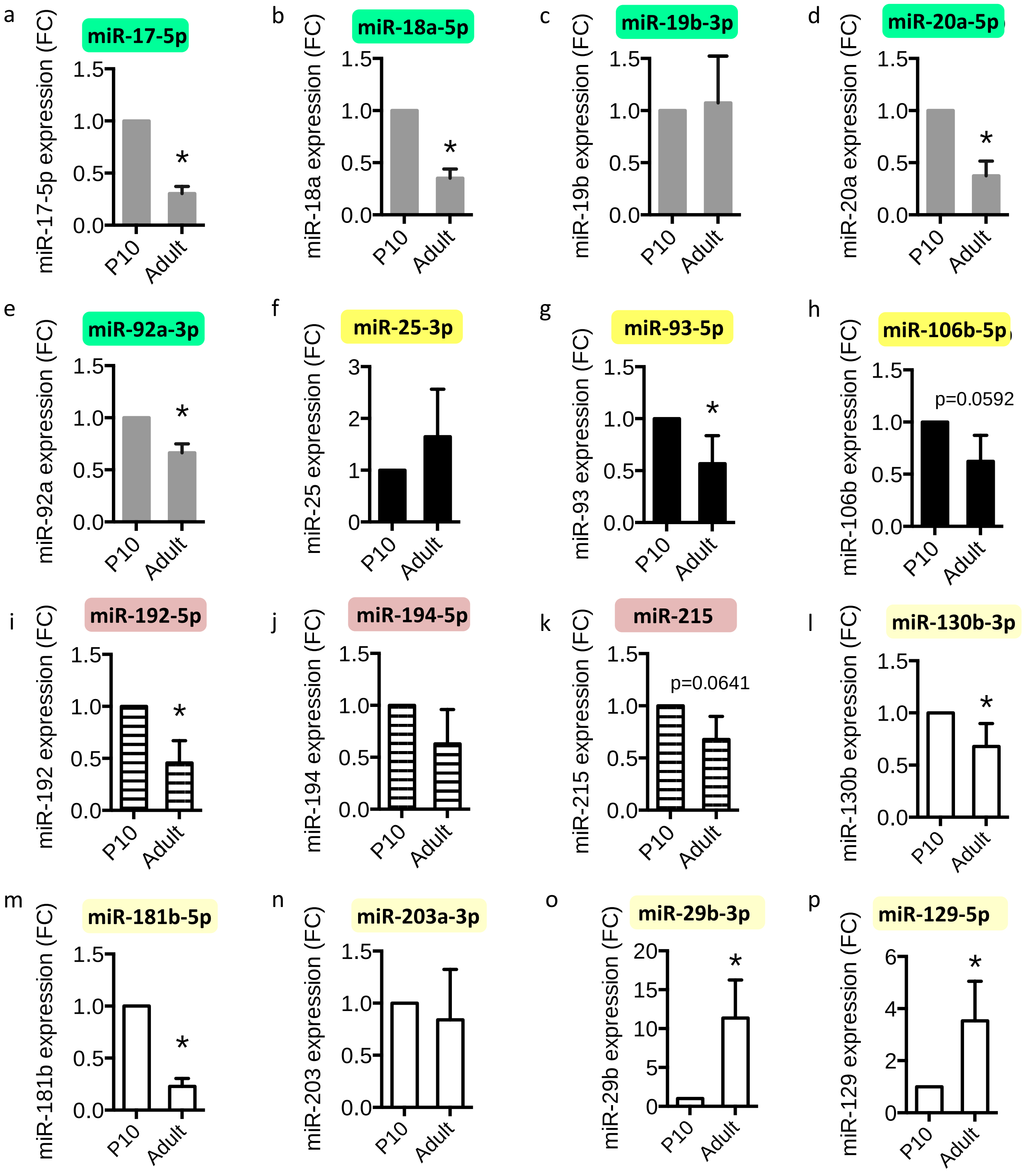

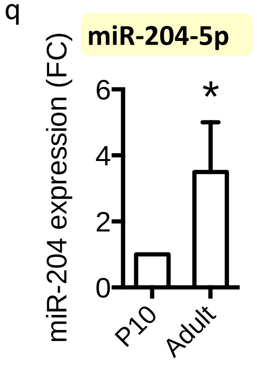

Supplementary Figure 6. microRNA expression changes during postnatal development occur in enriched-beta-cell population. **Beta-cells** were purified by **FACS and** miRNA levels were measured by qRT-PCR, normalized by U6 and expressed as fold change (FC). Data are presented as the mean ± SD and statistical significance versus P10 was analyzed by one-way ANOVA with test of mean: *P<0.05 (n=3 per group).

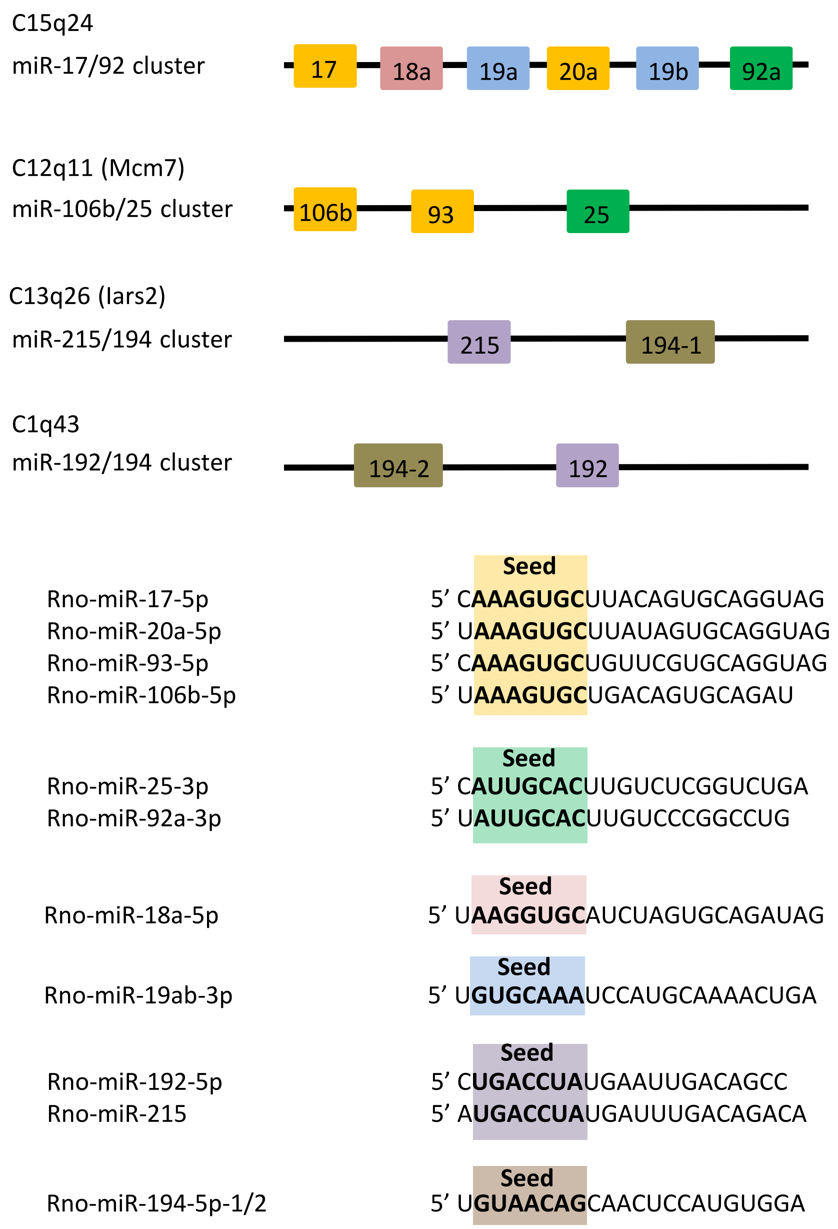

Supplementary Figure 7. A schematic view of the microRNA clusters identified in the microarray. The chromosomic locations of the miRNA cluster-encoding genes in rat are indicated. The individual miRNAs encoded from polycistonic genes are represented by boxes. miRNA sharing the same seed sequence are represented by boxes of the same colour. Seed sequences are shown in bold.

C13q21		**Seed**
Rno-miR-181a-5p	5’ A**ACAUUCA**ACGCUGUCGGUGAGU	
C13q21		
Rno-miR-181b-5p	5’ A**ACAUUCA**UUGCUGUCGGUGGGU	
C19q11		
Rno-miR-181c-5p	5’ A**ACAUUCA**ACCUGUCGGUGAGU	
C19q11		
Rno-miR-181d-5p	5’ A**ACAUUCA**UUGUUGUCGGUGGGU	

C4q21		**Seed**
Rno-miR-29a-3p	5’ U**AGCACCA**UCUGAAAUCGGUUA	
C4q21		
Rno-miR-29b-3p	5’ U**AGCACCA**UUUGAAAUCAGUGUU	
C1p11		
Rno-miR-29c-5p	5’ U**GACCGAU**UUCUCCUGGUGUUC	

Supplementary Figure 8. A schematic view of the microRNA families identified in the microarray. The chromosomic locations of the miRNA family-encoding genes in rat are indicated. Seed sequences are shown in bold.

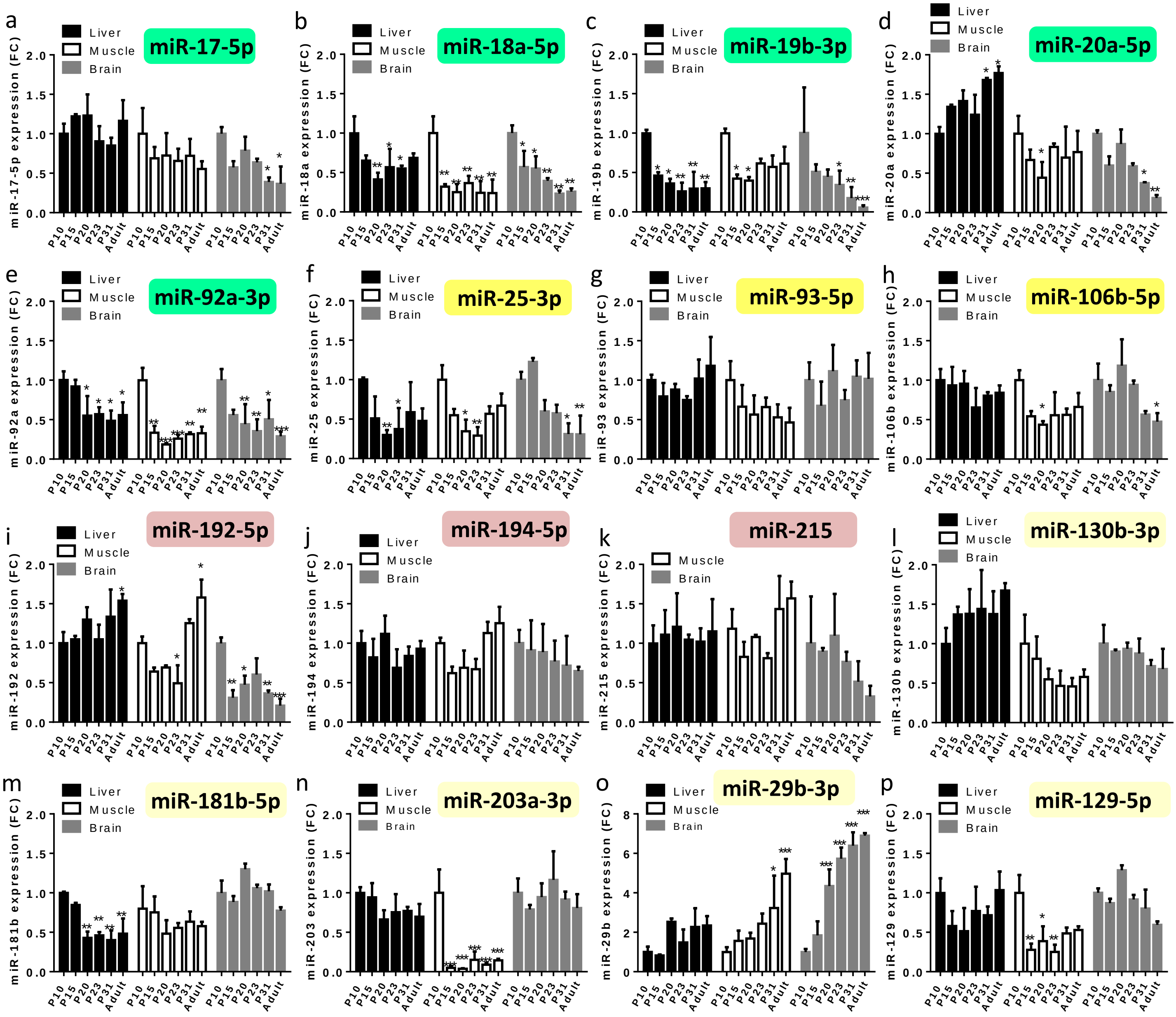

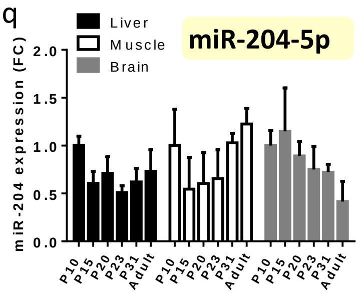

Supplementary Figure 9. miRNA expression profile in liver, muscle and brain during postnatal period. miRNA levels were measured by qRT-PCR, normalized by U6 and expressed as fold change (FC). Data are presented as the mean ± SD and statistical significance versus P10 was analyzed by one-way ANOVA with Dunnett post-hoc test: *P<0.05, **P<0.01, ***P<0.001 (n=3 per group).

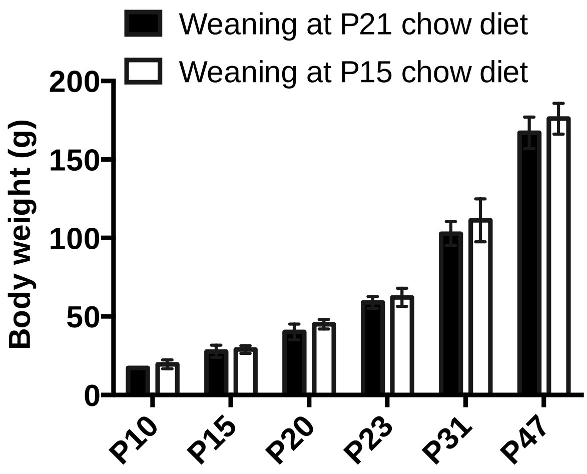

Supplementary Figure 10. Body weight of pups weaned at P21 (normal weaning) or P15 (premature weaning). n=4-8 per group.

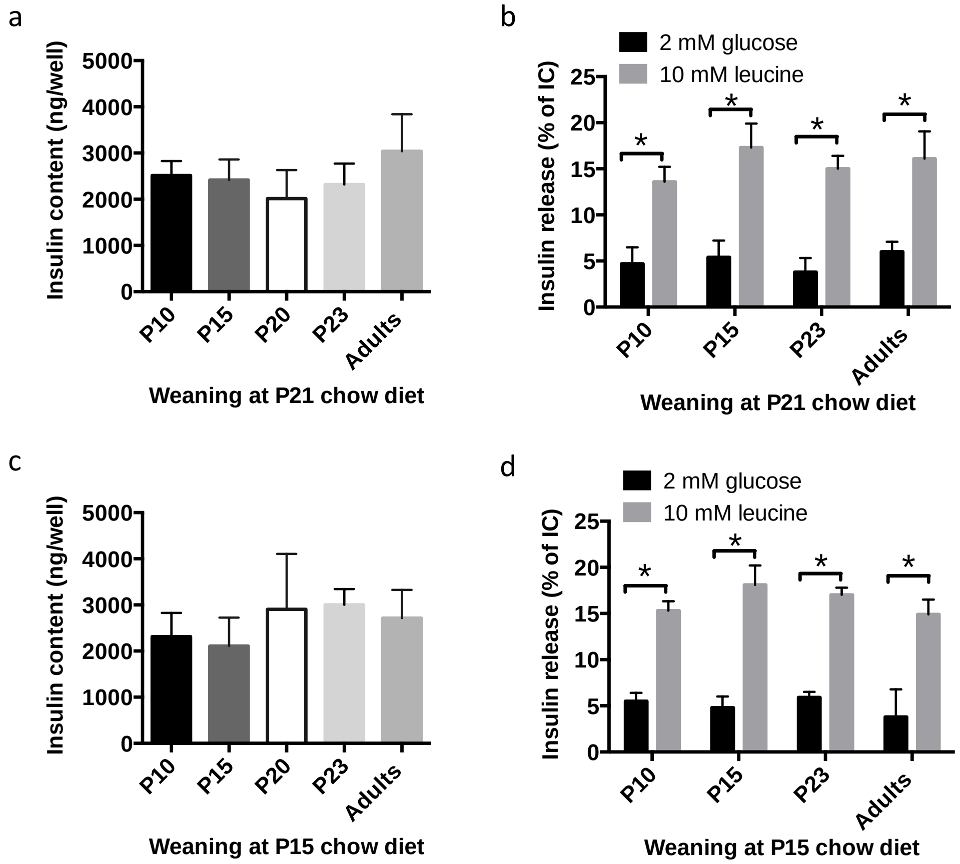

Supplementary Figure 11. Insulin secretion and content. (**a,c**) Insulin content and (**b,d**) insulin release after 45 min exposure to 2 mM glucose or 10 mM leucine (n=3-6) in rats weaned at P21 (top panel) or P15 (lower panel). Insulin contents and levels were measured by ELISA. Data are means ± SD (ANOVA, *<P0.05).

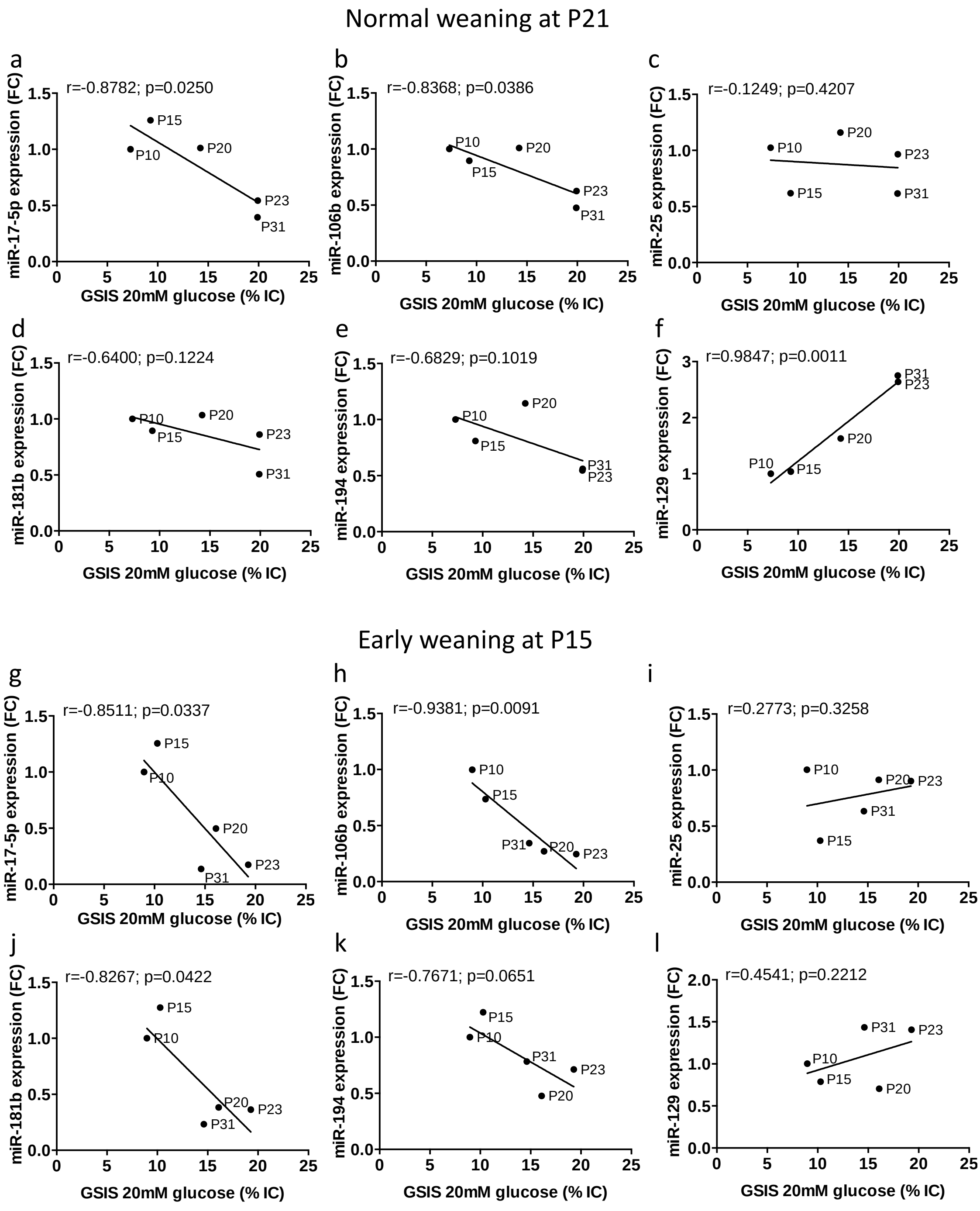

Supplementary Figure 12. Positive and negative correlations between miRNA levels and glucose-stimulated insulin secretion during time course. Rats were weaned at P21 (**a-f**) or at P15 (**g-l**). miRNA levels were measured by qRT-PCR, normalized by U6 and expressed as fold change (FC). Insulin release upon exposure of whole islets to 20 mM glucose for 45 min was assessed by ELISA, normalized by insulin content and expressed as fold change (FC). Data are the mean from 4 to 6 independent measurements. P-values ≤ 0.05 considered for significance. Correlations were established with Pearson correlation test.

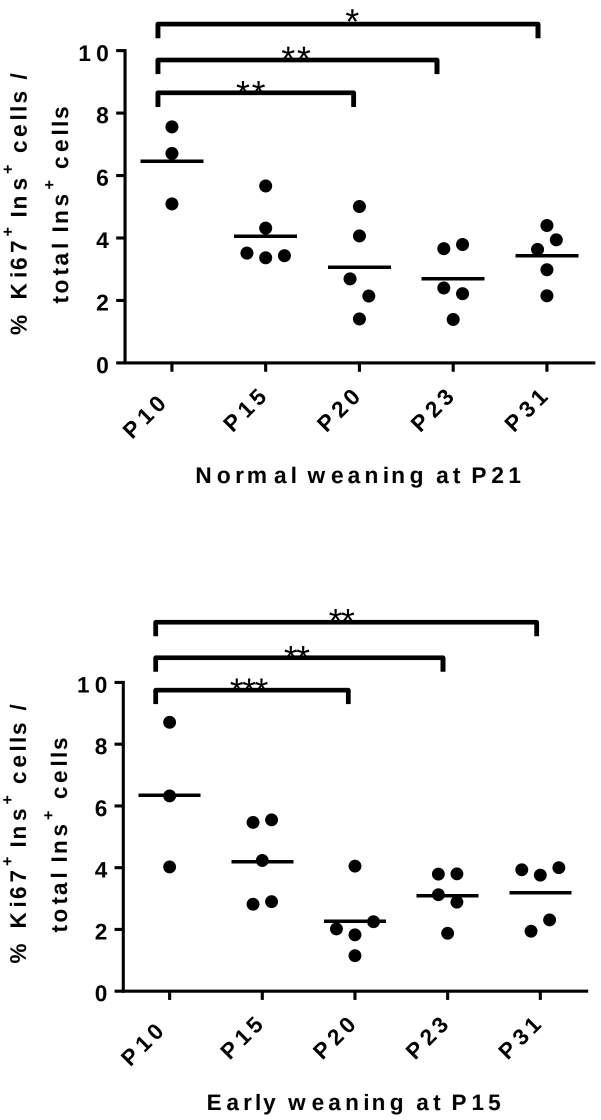

Supplementary Figure 13. Impact of premature weaning on β-cell proliferation. Proliferating Ki67-positive cells in rats weaned at P21 (top panel) or P15 (lower panel). Statistical differences from P10 was determined by ANOVA using Tukey's post-hoc test for multiple comparisons. *P<0.05, **P<0.01, ***P<0.001 (n=3-5 per group).

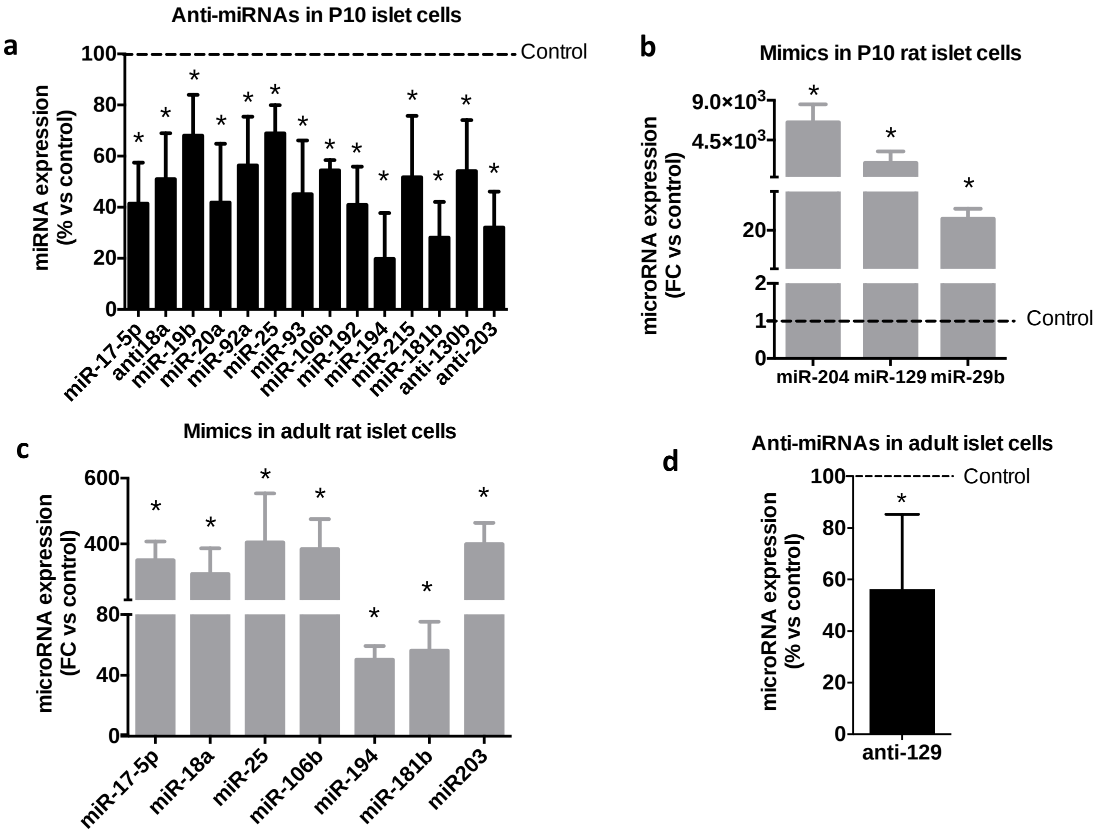

Supplementary Figure 14. Down- and up-regulation of microRNAs in dispersed rat islet cells. 10-day-old (**a,b**) and adult (**c,d**) rat islets were dissociated and transfected for 48h. To reduce the indicated miRNAs either in P10 (a) or in adult (**d**) islet cells, we used single-stranded anti-miRNAs or a scrambled anti-miR sequence as control. To increase the level of the indicated miRNAs either in P10 (**b**) or adult (**c**) islets cells, we used oligonucleotide mimics or an siRNA duplex against GFP as control. Results are expressed as fold changes *versus* control or as percentage of the control anti-miR ± SD (n=3-7). Statistical differences were determined by Student's t-test (*P<0.05).

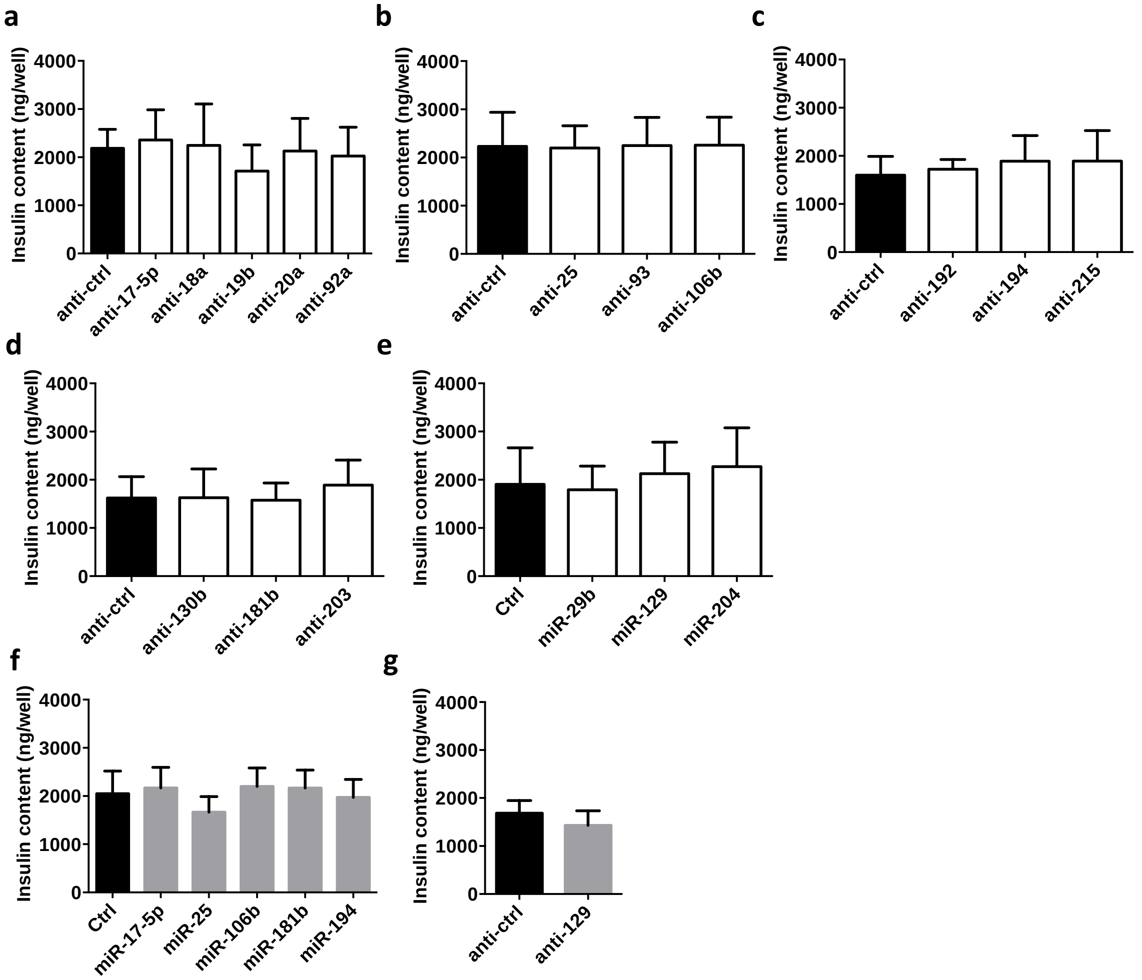

Supplementary Figure 15. Insulin content from dissociated P10 and adult rat islet cells. 10-day-old (**a-e**) and adult (**f,g**) rat islets were dispersed, transfected for 48h and incubated in the presence of 2 mM or 20 mM glucose for 45 min. Insulin contents were measured by ELISA (n=5-6). Data are means ± SD (ANOVA, *<P0.05).

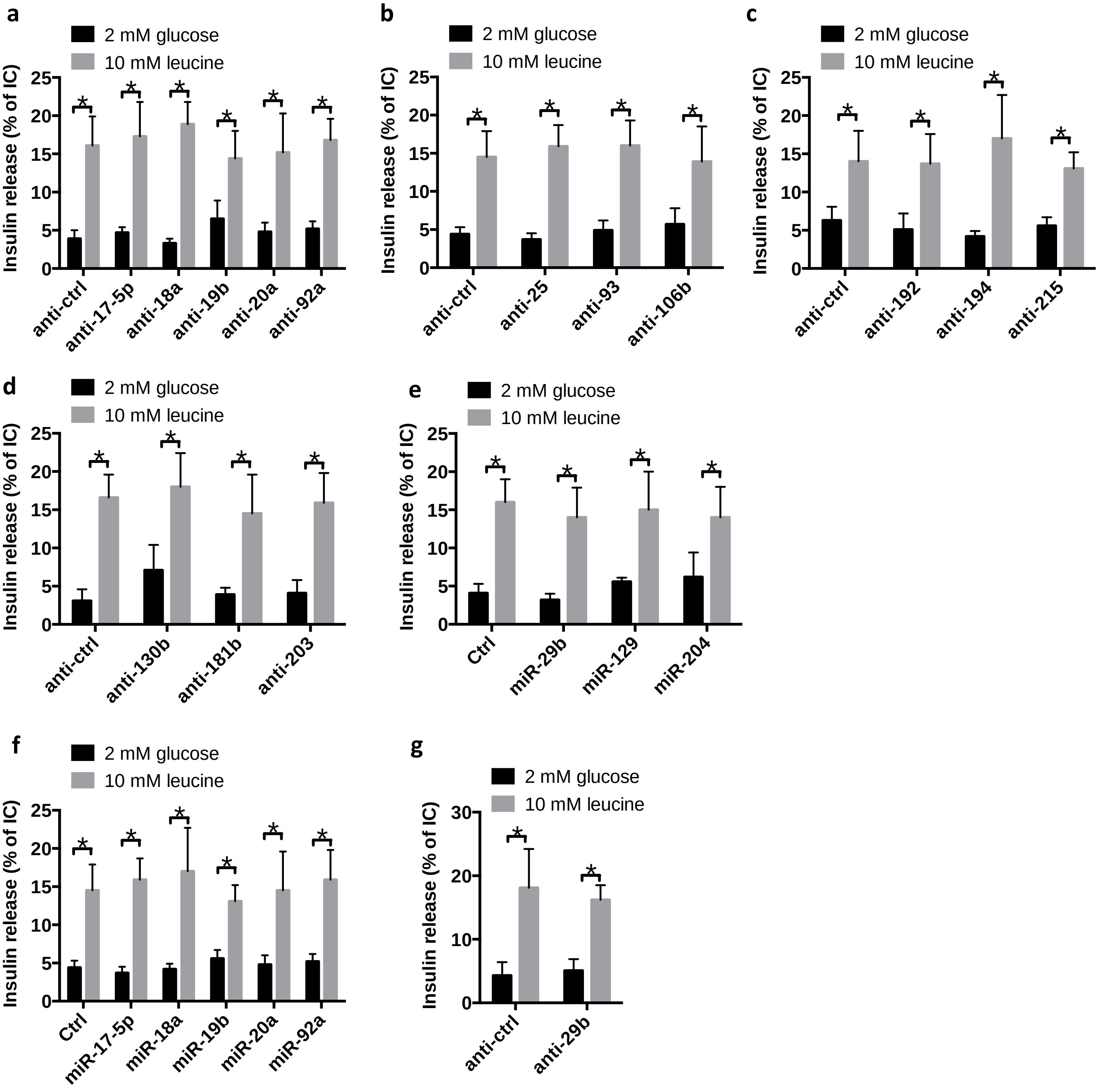

Supplementary Figure 16. Insulin secretion from dissociated P10 and adult rat islet cells. 10-day-old (**a-e**) and adult (**f,g**) rat islets were dispersed, transfected for 48h and incubated in the absence or the presence of 10 mM leucine for 45 min. Insulin levels and insulin contents were measured by ELISA (n=5-6). Data are means ± SD (ANOVA, *<P0.05).

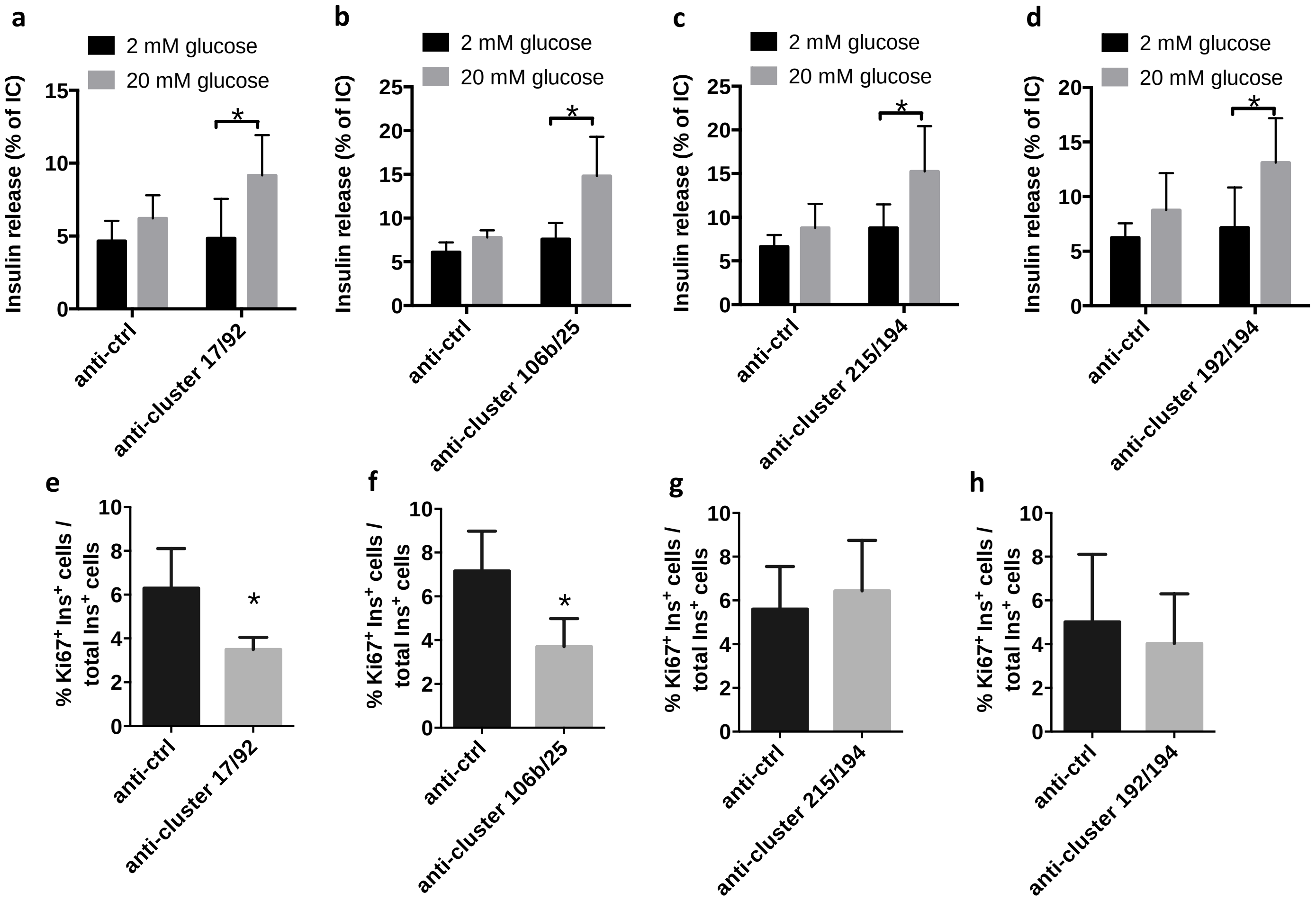

Supplementary Figure 17. Insulin secretion and β-cell proliferation from dissociated P10 rat islet cells. Dissociated P10 rat islet cells were transfected with a pool of the indicated antimiRs. Two days later, insulin release and content was assessed in P10 islet cells incubated at 2 or 20 mM glucose (n=4). The proliferating insulin+ cells from P10 rats was assessed by Ki67 staining (n=4). Data are means ± SD. Statistical significance versus anti-ctrl was assessed by ANOVA: *P<0.05.

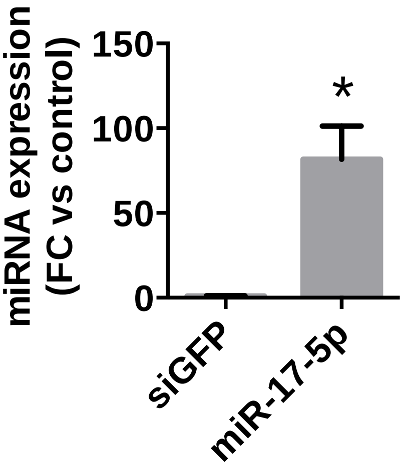

Supplementary Figure 18. miR-17-5p overexpression in dispersed rat islet cells. 10-day-old rat islets were dissociated and transfected for 48h. We used oligonucleotide mimics corresponding to the mature sequence of miR-17-5p or a siRNA duplex against GFP as a control. Results are expressed as fold change *versus* control (n=3). Data are presented as the mean ± SD and statistical differences were determined by ANOVA and Student's t-test (*P<0.05).

Supplementary Table 1. Enriched cell cycle pathway in adult versus 10-day-old rat islets defined by KEGG pathway analysis

Cell cycle-KEGG reference rno 04110 - Adult versus P10 rat islets								
**Gene symbol**	**Gene description**	**FC (array)**	**p-value**	**FC (qRT-PCR)**	**p-value**	**FC (RNA-seq)**	**Adj p-value**	**Adult FPKM (RNA-seq)**
Ccna2	Cyclin A2	-4.2	2.0E-04	-3.2	1.8E-02	below read cutoff		0.1
Ccnb1	Cyclin B1	-4.1	5.0E-03	-45.6	6.2E-03	-5.9	7.2E-06	1.6
Ccnb2	Cyclin B2	-3.7	1.4E-06	-	-	below read cutoff		2.4
Ccnd2	Cyclin D2	-5.1	3.1E-06	1.6	1.2E-02	1.3	2.2E-01	85.5
Ccne1	Cyclin E	-2.2	6.2E-05	-	-	below read cutoff		0.9
Ccnjl	Cyclin J-like	-4.8	6.6E-07	-	-	below read cutoff		0.1
Cdkn1a	Cyclin-dependent kinase inhibitor 1A (p21)	a	a	-1.1	1.6E-01	-1.6	1.6E-01	16.1
Cdkn1b	Cyclin-dependent kinase inhibitor 1B (p27)	2.0	4.2E-03	1.0	1.6E-01	-1.0	9.7E-01	20.3
Myc	Myelocytomatosis viral oncogene (c-Myc)	-6.5	2.4E-05	-4.3	2.6E-02	-2.9	3.1E-02	4.4
Cyld	Cylindromatosis	1.7	1.78E-04	1.6	3.2E-02	1.3	5.8E-01	5.7
Mcm7	Minichromosome maintenance deficient 7	-3.8	1.9E-04	-	-	-2.4	2.5E-03	7.8
Rbl2	Retinoblastoma-like 2 (Rbl2, p130)	1.6	7.69E-03	1.2	4.1E-01	1.2	8.1E-01	3.9
Pten	Phosphatase and tensin homolog	1.0	2.43E-01	2.1	1.1E-02	below read cutoff		3.1
Smad2	SMAD family member 2	-1.0	8.37E-01	-1.3	6.0E-02	-1.7	5.1E-01	5.0
Smad3	SMAD family member 3	a	a	-2.4	5.7E-02	below read cutoff		0.6
Smad4	SMAD family member 4	1.1	1.30E-01	-1.3	5.4E-02	-1.1	8.9E-01	17.5
Tgfb2	Transforming growth factor, beta 2	2.2	6.5E-04	-1.1	2.5E-01	below read cutoff		1.4
Tgfbr1	Transforming growth factor, beta receptor 1	2.2	1.3E-02	1.1	3.4E-01	-1.1	8.2E-01	3.5
Tgfbr2	Transforming growth factor, beta receptor 2	a	a	1.6	4.3E-02	-2.7	5.6E-02	2.4
Timp2	TIMP metallopeptidase inhibitor 2	-1.1	7.19E-01	1.0	5.0E-01	-3.1	3.6E-02	7.9
Timp3	TIMP metallopeptidase inhibitor 3	1.0	4.53E-04	1.0	2.7E-01	-1.5	5.8E-01	4.9

The fold change (FC) values reported from the array (n=3 per group) and confirmed by qRT-PCR (n=4 per group) and RNA-sequencing (n=3 per group). (a: absent; Adj-p value <0.05).

Supplementary Table 2. Enriched DNA replication pathway in adult versus 10-day-old rat islets defined by KEGG pathway analysis

DNA replication-KEGG reference Ko 03030 - Adult versus P10 rat islets								
**Gene symbol**	**Gene description**	**FC (array)**	**p-value**	**FC (qRT-PCR)**	**p-value**	**FC (RNA-seq)**	**Adj p-value**	**Adult FPKM (RNA-seq)**
Fen1	Flap structure-specific endonuclease 1	-2.6	5.2E-03	-	-	-2.2	1.0E-02	4.4
Lig1	Ligase I, DNA, ATP-dependent	-2.8	1.4E-04	-	-	-2.5	7.2E-04	5.5
Pola2	Polymerase (DNA directed), alpha 2	-3.7	1.1E-03	-	-	below read cutoff		1.2
Pold2	Polymerase (DNA directed), delta 2	-4.0	1.2E-06	-	-	below read cutoff		3.8
Mcm2	Minichromosome maintenance deficient 2	-3.6	3.0E-04	-	-	-3.0	5.6E-06	5.0
Mcm5	Minichromosome maintenance deficient 5	-3.3	4.4E-02	-	-	-2.9	3.9E-03	3.8
Mcm6	Minichromosome maintenance deficient 6	-3.5	5.6E-05	-	-	-2.7	1.2E-03	3.5
Mcm7	Minichromosome maintenance deficient 7	-3.8	1.9E-04	-	-	-2.4	2.5E-03	7.8
Rfc3	Replication factor C (activator 1) 3	-3.1	1.1E-05	-	-	-3.0	5.8E-03	6.0
Rfc4	Replication factor C (activator 1) 4	-3.7	7.8E-07	-	-	below read cutoff		2.5

The fold change (FC) values reported from the array (n=3 per group) and confirmed by qRT-PCR (n=4 per group) and RNA-sequencing (n=3 per group). (a: absent; Adj-p value <0.05).

Supplementary Table 3. Enriched insulin secretion pathway in adult versus 10-day-old rat islets defined by KEGG pathway analysis

Insulin secretion-KEGG reference rno 04911 - Adult versus P10 rat islets								
**Gene symbol**	**Gene description**	**FC (array)**	**p-value**	**FC (qRT-PCR)**	**p-value**	**FC (RNA-seq)**	**Adj p-value**	**Adult FPKM (RNA-seq)**
Adcy8	Adenylate cyclase	4.2	5.3E-07	-	-	below read cutoff		0.3
Camk2b	ium/calmodulin-dependent protein kinase II,	3.8	5.3E-04	-	-	1.1	6.7E-01	90.3
Gck	Glucokinase	5.9	3.8E-05	1.3	8.7E-02	-1.1	5.9E-01	20.8
Glp1r	Glucagon-like peptide 1 receptor	9.5	3.8E-05	-	-	1.8	9.2E-03	72.7
Gipr	Gastric inhibitory polypeptide receptor	2.4	6.5E-05	-	-	1.6	2.5E-02	119.4
Slc2a2	Solute carrier family 2, member 2 (Glot2)	9.7	4.9E-05	8.2	1.9E-03	2.2	2.3E-06	326.7
Stk11	Serine/threonine kinase 11 (Lkb1)	a	a	-1.8	2.2E-02	1.0	8.8E-01	30.8
Sirt1	Sirtuin 1	a	a	1.7	2.9E-02	a	a	not in Rnor_5.0 database
Ucn3	Urocortin 3	6.8	1.5E-04	12.0	7.8E-04	1.9	1.3E-02	357.3
VAMP2	Vesicle-associated membrane protein 2	2.9	1.3E-06	-	-	1.8	8.6E-03	92.4

The fold change (FC) values reported from the array (n=3 per group) and confirmed by qRT-PCR (n=4 per group) and RNA-sequencing (n=3 per group). (a: absent; Adj-p value <0.05).

Supplementary Table 4. Enriched glycolysis/gluconeogenesis pathway in adult versus 10-day-old rat islets defined by KEGG pathway analysis

Glycolysis/Gluconeogenesis-KEGG reference rno 00010 - Adult versus P10 rat islets								
**Gene symbol**	**Gene description**	**FC (array)**	**p-value**	**FC (qRT-PCR)**	**p-value**	**FC (RNA-seq)**	**Adj p-value**	**Adult FPKM (RNA-seq)**
Aldob	Aldolase B	-44.4	4.7E-08	-30.5	1.5E-02	below read cutoff		0.0
Aldoa	Aldolase A	1.9	2.7E-03	-	-	1.6	1.9E-02	991.4
Eno2	Enolase 2, gamma	5.3	7.2E-05	1.6	1.3E-02	below read cutoff		43.5
Gapdh	Glyceraldehyde-3-phosphate dehydrogenase	1.4	3.1E-02	1.1	3.3E-01	1.3	3.4E-01	1619.3
G6pc	Glucose-6-phosphatase, catalytic	-114.6	5.2E-07	-31.0	6.5E-03	below read cutoff		0.4
G6pi	Glucose-6-phosphate isomerase	1.2	2.1E-01	2.1	2.0E-02	a	a	not in Rnor_5.0 database
Gck	Glucokinase	5.9	3.8E-05	1.3	8.7E-02	-1.1	5.9E-01	20.8
Glud1	Glutamate dehydrogenase 1	-2.2	2.5E-04	-1.1	4.5E-01	-1.0	8.7E-01	49.4
Gpd2	erol-3-phosphate dehydrogenase 2, mitochon	1.1	6.4E-04	2.0	2.4E-02	1.5	5.2E-01	7.2
Ldha	Lactate dehydrogenase A	-3.9	1.5E-04	-3.0	5.1E-02	-3.5	3.8E-04	55.1
Mdh1	Malate dehydrogenase 1, NAD (soluble)	a	a	3.1	1.5E-02	1.9	5.9E-04	362.3
Mdh2	Malate dehydrogenase 2, NAD (soluble)	1.3	6.9E-04	1.0	4.2E-01	1.1	8.5E-01	181.7
Pc	Pyruvate carboxylase	2.3	1.1E-04	3.8	3.9E-03	4.0	6.1E-13	44.3
Pfkm	Phosphofructokinase, muscle	3.3	2.6E-05	2.0	3.9E-02	1.2	3.2E-01	21.8
Pfkp	Phosphofructokinase, platelet	1.5	7.1E-04	3.8	1.2E-03	1.8	1.8E-02	17.5
Pck1	hosphoenolpyruvate carboxykinase 1 (PEPCK	-1060.6	1.3E-07	-719.6	1.3E-02	below read cutoff		0.0
Pck2	hosphoenolpyruvate carboxykinase 2 (PEPCK	-2.3	1.3E-05	-1.3	1.6E-01	1.0	8.9E-01	50.5
Pdha1	Pyruvate dehydrogenase alpha 1	1.1	7.3E-01	2.2	3.0E-02	-1.1	6.8E-01	25.7
Pgam1	Phosphoglycerate mutase 1	2.3	3.5E-06	1.7	2.2E-02	1.4	1.3E-01	169.8
Pgam2	Phosphoglycerate mutase 2	2.2	3.9E-04	-	-	below read cutoff		2.2
Pkm2	Pyruvate kinase, muscle	4.5	1.8E-04	2.0	5.7E-01	a	a	not in Rnor_5.0 database
Slc16a1	family 16 (monocarboxylate transporter), me	-5.6	5.7E-05	-2.8	9.4E-03	a	a	1.1

The fold change (FC) values reported from the array (n=3 per group) and confirmed by qRT-PCR (n=4 per group) and RNA-sequencing (n=3 per group). (a: absent; Adj-p value <0.05).

Supplementary Table 5. Global microRNA profiling of adult versus newborn rat islets. 311 microRNAs were analyzed by microarray and 68 were significantly change > 2 fold. 30 were up-regulated (left panel) and 38 were down-regulated (right panel) in 3-month-old rat islets compared to 10-day-old rat islets. Data are expressed in fold change (FC) and mean are given in Log2 scale and are the mean ± SD of 4 individual rats (p<0.05)

Up-regulated microRNAs > 2 fold				
**microRNA ID**	**Fold change Adult vs P10**	**adj.P.Value**	**Mean P10 (Log2)**	**Mean adult (Log2)**
rno-miR-29b-3p	8.6	2.53E-04	10.60	13.71
rno-miR-344a-3p	6.0	9.04E-04	4.58	7.16
rno-miR-129-5p	5.7	4.60E-03	5.86	8.36
rno-miR-132-3p	5.4	3.11E-03	4.67	7.11
rno-miR-29c-3p	5.4	1.84E-04	10.00	12.44
rno-miR-129-2-3p	5.0	1.44E-03	6.61	8.93
rno-miR-129-1-3p	4.9	3.1E-04	5.89	8.19
rno-miR-384-5p	4.8	3.8E-03	8.21	10.48
rno-miR-383-5p	4.6	1.9E-04	3.49	5.68
rno-miR-29a-3p	4.5	1.8E-04	11.12	13.28
rno-miR-29c-5p	4.1	2.0E-04	5.64	7.68
rno-miR-204-5p	3.8	3.7E-04	7.51	9.44
rno-miR-325-3p	3.7	1.3E-02	6.43	8.32
rno-miR-22-5p	3.5	5.8E-04	4.16	5.96
rno-miR-153-3p	3.4	7.9E-03	7.85	9.62
rno-miR-483-5p	3.1	4.4E-03	6.92	8.55
rno-miR-7a-2-3p	3.0	1.2E-02	5.71	7.30
rno-miR-598-3p	3.0	1.7E-02	6.22	7.79
rno-miR-384-3p	2.9	7.3E-04	3.74	5.30
rno-miR-455-5p	2.9	3.1E-03	6.40	7.96
rno-miR-195-5p	2.9	8.4E-04	8.10	9.65
rno-miR-451-5p	2.8	8.5E-03	7.29	8.77
rno-miR-22-3p	2.6	1.3E-03	10.78	12.16
rno-miR-3546	2.5	3.9E-02	4.34	5.64
rno-miR-183-5p	2.4	2.8E-03	8.59	9.82
rno-miR-325-5p	2.3	2.4E-03	4.10	5.31
rno-miR-497-5p	2.2	2.5E-03	8.22	9.38
rno-miR-98-5p	2.2	8.9E-04	8.90	10.03
rno-miR-495	2.0	2.4E-03	5.72	6.73
rno-miR-144-3p	2.0	2.7E-02	4.64	5.64

Down-regulated microRNAs > 2 fold				
microRNA ID	Fold change Adult vs P10	adj.P.Value	Mean P10 (Log2)	Mean adult (Log2)
rno-miR-215	-139.2	5.82E-04	9.62	2.50
rno-miR-194-5p	-9.5	8.70E-03	10.16	6.91
rno-miR-203a-3p	-6.9	2.58E-03	7.60	4.81
rno-miR-192-5p	-6.8	1.37E-02	10.85	8.09
rno-miR-130b-3p	-5.3	8.92E-04	7.96	5.57
rno-miR-181b-5p	-5.1	3.86E-03	7.68	5.35
rno-miR-181d-5p	-4.7	7.4E-04	7.18	4.94
rno-miR-19b-3p	-4.5	6.0E-04	11.30	9.13
rno-miR-181a-5p	-4.4	3.1E-03	9.54	7.41
rno-miR-92a-3p	-4.3	4.5E-03	10.43	8.31
rno-miR-17-5p	-4.2	2.3E-04	8.01	5.94
rno-miR-20b-5p	-3.8	2.6E-04	8.65	6.72
rno-miR-181c-5p	-3.8	3.3E-04	7.82	5.91
rno-miR-503-5p	-3.8	3.1E-03	4.86	2.95
rno-miR-18a-5p	-3.7	2.0E-04	6.44	4.56
rno-miR-20a-5p	-3.6	3.7E-04	9.50	7.64
rno-miR-192-3p	-3.5	2.2E-02	4.03	2.23
rno-miR-19a-3p	-3.5	3.3E-04	8.56	6.77
rno-miR-322-3p	-3.3	9.5E-04	5.30	3.57
rno-miR-542-5p	-3.2	6.0E-04	5.67	3.99
rno-miR-1949	-3.0	1.2E-02	7.46	5.87
rno-miR-542-3p	-3.0	1.3E-03	5.72	4.14
rno-miR-93-5p	-2.8	2.7E-04	8.48	6.97
rno-miR-199a-5p	-2.8	2.5E-04	8.83	7.33
rno-miR-450a-5p	-2.8	1.8E-04	6.92	5.45
rno-miR-199a-3p	-2.7	2.5E-04	10.43	8.98
rno-miR-214-3p	-2.7	3.1E-04	8.67	7.22
rno-miR-675-3p	-2.6	7.9E-03	4.63	3.25
rno-miR-184	-2.5	2.9E-02	4.94	3.64
rno-miR-378a-3p	-2.4	1.4E-02	7.70	6.42
rno-miR-218a-5p	-2.3	2.0E-04	7.78	6.58
rno-miR-25-3p	-2.3	8.5E-03	10.00	8.81
rno-miR-15b-5p	-2.3	2.5E-04	10.96	9.79
rno-miR-106b-5p	-2.2	5.1E-04	10.05	8.92
rno-miR-301b-3p	-2.1	6.0E-04	4.22	3.13
rno-miR-365-3p	-2.1	1.6E-02	7.91	6.83
rno-miR-181a-1-3p	-2.1	8.9E-04	5.02	3.95
rno-miR-20a-3p	-2.0	5.1E-04	4.26	3.26

Supplementary Table 6. Predicted miRNA targets. Predicted targeted genes related to glucose metabolism, cell proliferation and insulin secretion are reported for the 8 miRNAs (miR-17-5p, miR-18a, miR-25, miR-106b, miR-181b, miR-194, miR-203, miR-129) that we identified to significantly play a role in postnatal β-cell maturation. miR-17-5p, miR-106b and miR-181b are highlighted in green and purple, respectively. and miRSystem algorithms which integrates miRNA target gene predictions in human and rats from DIANA, miRanda, miRBridge, PicTar, PITA, rna22, were used.

Predicted target mRNAs			
**Gene name**	**Gene description**	**miRNA**	**Category**
Birc5 (Survivin)	Baculoviral IAP repeat-containing 5	miR-203	Mitogen and anti-apoptotic
Ccnd2	Cyclin D2	miR-17-5p	Mitogen
		miR-106b	
		miR-18a	
Cdkn1a (p21)	Cyclin-dependent kinase inhibitor 1A	miR-17-5p	Tumor supressor
		miR-106b	
Cdkn1b (p27)	Cyclin-dependent kinase inhibitor 1B	miR-181b	Tumor supressor
Cdkn1c (p57)	Cyclin-dependent kinase inhibitor 1C	miR-129	Tumor supressor
Cyld	Cylindromatosis (turban tumor syndrome)	miR-17-5p	
		miR-106b	
		miR-181b	
G6pc	Glucose-6-phosphatase	miR-203	Gluconeogenesis
Got2	Glutamic-oxaloacetic transaminase 2, mitochondrial	miR-194	TCA cycle
Gpd2	Glycerol-3-phosphate dehydrogenase 2, mitochondrial	miR-17-5p	Mitochondrial shuttle
		miR-106b	
		miR-181b	
Hnf1b	HNF1 homeobox B	miR-25	Pancreas development
		miR-194	
Idh1	Isocitrate dehydrogenase 1	miR-25	TCA cycle
Irs2	Insulin receptor substrate 2	miR-25	Mitogen, anti-apoptotic, insulin secretion regulator
		miR-181b	
Ldha	Lactate dehydrogenase A	miR-203	Aerobic glycolysis
Mdh1	Malate dehydrogenase 1	miR-181b	Mitochondrial shuttle
p130 (Rbl2)	Retinoblastoma-like 2	miR-17-5p	Tumor supressor
		miR-106b	
Pfkp	Phosphofructokinase, platelet	miR-17-5p	Glycolysis
		miR-106b	
Pgam1	Phosphoglycerate mutase 1	miR-25	Glycolysis
Pten	Phosphatase and tensin homolog	miR-17-5p	Tumor supressor
		miR-106b	
		miR-181b	
		miR-18a	
		miR-25	

Predicted target mRNAs			
**Gene name**	**Gene description**	**miRNA**	**Category**
Tcf7l2	Transcription factor 7-like 2	miR-181b	Insulin secretion and β-cell regeneration regulator
		miR-194	
Tgfbr1	Transforming growth factor, beta receptor1	miR-181b	Tumor supressor
Tgfbr2	Transforming growth factor, beta receptor2	miR-17-5p	Tumor supressor
		miR-106b	
		miR-181b	
Timp2	TIMP metallopeptidase inhibitor 2	miR-17-5p	Tumor supressor
		miR-106b	
Timp3	TIMP metallopeptidase inhibitor 3	miR-181b	Tumor supressor
Sirt1	Sirtuin 1	miR-181b	Proliferation, survival and insulin secretion regulator
		miR-194	
Slc1a3	Solute carrier family 1 (glial high affinity glutamate transporter), member 3	miR-194	Glutamate aspartate transporter
Slc2a2	Solute carrier family 2 (facilitated glucose transporter), member 2	miR-17-5p	Glucose transporter
		miR-106b	
		miR-194	
Slc2a3	Solute carrier family 2 (facilitated glucose transporter), member 3	miR-25	Glucose transporter
Smad2	SMAD family member 2	miR-18a	Tumor supressor
		miR-181b	
Smad4	SMAD family member 4	miR-17-5p	Tumor supressor
		miR-106b	
Sox4	SRY (sex determining region Y)-box 4	miR-129	Pancreas development
Stk11 (Lkb1)	Serine/threonine kinase 11	miR-17-5p	Tumor supressor and insulin secretion regulator
		miR-106b	

Supplementary Table 7. Predicted miRNA targets for miR-29b-3p. Predicted targeted genes related to glucose metabolism, cell proliferation and insulin secretion are reported for miR-29b. miRWalk, miRSystem and TargetScan algorithms were used.

Predicted target mRNAs for miRNA-29b		
**Gene name**	**Gene description**	**Category**
G6pc	Glucose-6-phosphatase, catalytic subunit	Gluconeogenesis
Mafb	v-maf avian musculoaponeurotic fibrosarcoma oncogene homolog B	Transcription factor involves in β-cell development and function
Pdgfra	Platelet-derived growth factor receptor, alpha polypeptide	Cell proliferation
Pdgfrb	Platelet-derived growth factor receptor, beta polypeptide	Cell proliferation
Rest	RE1-silencing transcription factor	Neuronal transcriptional repressor
Slc16a1 (=Mct1)	Solute carrier family 16, member 1 (monocarboxylic acid transporter1)	Pyruvate/lactate/ketone bodies transporter

Supplementary Table 8. List of gene ontology functions significantly enriched in the increased RISC Incorporation Ratio fraction of transcripts upon miR-17-5p overexpression in 10-day-old rat islet cells.

	No. of genes	Benjamini p-values
regulation of cell proliferation	11	0.02
regulation of macromolecule metabolic process	10	0.04
regulation of molecular function	9	0.03
regulation of cellular protein metabolic process	8	0.03
heart development	6	0.01
leukocyte activation	6	0.02
cell activation	6	0.03
regulation of protein modification process	6	0.04
mitotic cell cycle	6	0.01
regulation of binding	5	0.01
regulation of protein metabolic process	5	0.03
posttranscriptional regulation of gene expression	5	0.04
regulation of protein stability	4	0.00
regulation of ubiquitin-protein ligase activity during mitotic cell cycle	4	0.01
regulation of ubiquitin-protein ligase activity	4	0.01
regulation of ligase activity	4	0.01
regulation of protein ubiquitination	4	0.02
muscle system process	4	0.08
monocarboxylic acid transport	3	0.04
regulation of cell cycle	3	0.05

Supplementary Table 9. Primer sequences.

Genes	Forward primer	Reverse primer	Species
18S	5'-GGAATTATTCCCCATGAACG-3'	5'-GGCCTCACTAAACCATCCAA-3'	R, M, H
Aldob	5'-CCCTTGGCTGTACCTGTCAT-3'	5'-TGCCTTCGGTTCTCTTCAGT-3'	R
Ccna2	5'-GAGAATGTCAACCCCGAAAA-3'	5'-GGGACGTGCTCATCGTTTAT-3'	R
Ccnb1	5'-AATTGGAGAGGTCGATGTGG-3'	5'-CTCCGTGTGGGACAGGTAGT-3'	R
Ccnd2	5'-TTACCTGGACCGTTTCTTGG-3'	5'-TGCTCAATGAAGTCGTGAGG-3'	R, M, H
Cdkn1a	5'-TCAGTGGACCAGAAGGGAAC-3'	5'-GGTCCCCATCCCAGATAAGT-3'	R
Cdkn1b	5'-CAGAATCATAAGCCCCTGGA-3'	5'-TCTGACGAGTCAGGCATTTG-3'	R, M
C-myc	5'-CGAGCTGAAGCGTAGCTTTT-3'	5'-CTCGCCGTTTCCTCAGTAAG-3'	R
Cyld	5'-AGGAAAGGAGACCTCCCAAA-3'	5'-GCAGGGTCTTCTGCAACTTC-3'	R
Eno2	5'-GTGGACCACATCAACAGCAC-3'	5'-TGAGCAATGTGGCGATAGAG-3'	R, M
G6pc	5'-ACCCTGGTAGCCCTGTCTTT-3'	5'-GGGCTTTCTCTTCTGTGTCG-3'	R
G6pi	5'-CACTGCATGTGGGTTTTGAC-3'	5'-GCTGGAAATAGGCAGCAAAG-3'	R
Gapdh	5'-AGACAGCCGCATCTTCTTGT-3'	5'-CTTGCCGTGGGTAGAGTCAT-3'	R
Gck	5'-TTTGCAACACTCAGCCAGAC-3'	5'-ATTTGTTGGTGCCCAGAGTC-3'	R
Glud1	5'-GGCGTCCCTAATGACCTACA-3'	5'-CCTATGGTGCTGGCATAGGT-3'	R
Gpd2	5'-CCGACTGACTCCGTTCTAGC-3'	5'-TACGCCAGGCTTACTTGCTT-3'	R
Irs2	5'-CCACACACCTGTCCTCATTG-3'	5'-TAATCCGCTTTGCCAAAATC-3'	R
Ldha	5'-AGACTTGGCCGAGAGCATAA-3'	5'-CTGCAGCTCCTTCTGGATTC-3'	R, M
Mafb	5'-TATTCCAAGGAGTCGCCAAG-3'	5'-CTGAGAGCCAGTGTTCACCA-3'	R
Mdh1	5'-GAAGCCCTCAAAGACGACAG-3'	5'-CGACAGGGAACGAGTAGAGC-3'	R
Mdh2	5'-TTGTCTTCTCCCTGGTGGAC-3'	5'-CAAAGTCCTCGCCTTTCTTG-3'	R, M
P85a	5'-ACCAGTGTTGACCCTTCCTG-3'	5'-GGCGAGATAGCGTTTGAAAG-3'	R
P130 (Rbl2)	5'-TAAGTCACTCCTGGCCTGCT-3'	5'-CTTGGTTTTCCATGCCAAGT-3'	R
Pc	5'-AGATGCACTTCCATCCCAAG-3'	5'-CCTTGGTCACGTGAACCTTT-3'	R
Pck1	5'-CCCAGGAGTCACCATCACTT-3'	5'-TTCGTAGACAAGGGGGACCA-3'	R
Pck2	5-'GCACGGGTAGAAAGCAAGAC-3'	5'-CATGCATCCTGGGAATCTCT-3'	R, M
Pdha1	5'-AGGAACACTGGGTGATCTGG-3'	5'-ACGGTAAGAGAGCCTGCAAA-3'	R
Pdgfra	5'-CTTGCAATCCGTCAGTAGCA-3'	5'-ACGTCCTCGGCTAGGGTTAT-3'	R
Pdgfrb	5'-TGTTCGTGCTATTGCTCCTG-3'	5'-TGTCAGCACACTGGAGAAGG-3'	R

Rat (R); Mouse (M); Human (H)

Genes	Forward primer	Reverse primer	Species
Pfkm	5’-AGGTGACCAAAGACGTGACC-3’	5’-TGGATAAGGCCAATCCTCAC-3’	R, M
Pfkp	5’-TTGACTGCAGGAAGAACGTG-3’	5’-TCCTTAGCTCTGCCACTGGT-3’	R
Pgam1	5’-CTCAGGGCAAGGTGAAGAAG-3’	5’-GGTGCCGGGGATAAAATACT-3’	R, M
Pkm2	5’-CCTATCCATTAGGCCAGCAA-3’	5’-TTTCCAATCCTGCATTCCTC-3’	R
Pten	5’-ACACCGCCAAATTTAACTGC-3’	5’-TACACCAGTCCGTCCTTTCC-3’	R
Rest	5’-ACAACGGGCCTAAACCTCTT-3’	5’-TCAGCTTCACTTTGGACACG-3’	R
Sirt1	5’-CCAGATCCTCAAGCCATGTT3-’	5’-GATCCTTTGGATTCCTGCAA-3’	R
Slc2a2 (Glut2)	5’-CAATTTCATCATCGCCCTCT-3’	5’-TGCAGCAATTTCGTCAAAAG-3’	R
Slc16a1 (Mct1)	5’-TATGCCGGAGGTCCTATCAG-3’	5’-AGTTGAAAGCAAGCCCAAGA-3’	R, M
Stk11 (Lkb1)	5’-CCTGCAGAGAAAACCCAGAG-3’	5’-GCAGCTTCAAGTTTCCCAAG-3’	R, M
Smad2	5’CCAGGTCTCTTGATGGTCGT-3’	5’-TCTCCACCCTCTGGTAGTGG-3’	R
Smad3	5’-CTTGGTGCAGAGACCTGTCA-3’	5’-CAGGTGGGATCCTATGTGCT-3’	R
Smad4	5’-TCGATTCAAACCATCCAACA-3’	5’-GCCCTGAAGCTATCTGCAAC-3’	R, M
Tcf7l2	5’-GTCGGCTCACTCCATAGCTC-3’	5’-TTCCGGGCTAGCTCGTAGTA-3’	R, M, H
Tgfb2	5’-AATCCTAGCCAGGGACGTTT-3’	5’-TGCAGGAGCAAAAAGGTTCT-3’	R
Tgfbr1	5’-TTTGAGGAGGGCAGCTTTTA-3’	5’-ACCAGTGAGGAGACCCAATG-3’	R
Tgfbr2	5’-TCACTAGGCACGTCATCAGC-3’	5’-AGGACAACCCGAAGTCACAC-3’	R
Timp2	5’-CAAGTTCTTTGCCTGCATCA-3’	5’-TCCAGGAAGGGATGTCAAAG-3’	R
Timp3	5’-TGTACACCCCAGCCTCTTTC-3’	5’-CTTCTCGCCAAGACCTCAAC-3’	R
Ucn3	5’-CTTCAGTCGCCTCAACACAG-3’	5’-GCTGTGCTTGGGATTGGTAT-3’	R

Rat (R); Mouse (M); Human (H)

Mature miRNA sequences.

Name	Mature miRNA sequences
rno-miR-106b-5p	5'-UAAAGUGCUGACAGUGCAGAU-3'
rno-miR-129-5p	5'-CUUUUUGCGGUCUGGGCUUGC-3'
rno-miR-130b-3p	5'-CAGUGCAAUGAUGAAAGGGCAU-3'
rno-miR-17-5p	5'-CAAAGUGCUUACAGUGCAGGUAG-3'
rno-miR-18a-5p	5'-UAAGGUGCAUCUAGUGCAGAUAG-3'
rno-miR-181b-5p	5'-AACAUUCAUUGCUGUCGGUGGGU-3'
rno-miR-19b-3p	5'-UGUGCAAAUCCAUGCAAAACUGA-3'
rno-miR-192-5p	5'-CUGACCUAUGAAUUGACAGCC-3'
rno-miR-194-5p	5'-UGUAACAGCAACUCCAUGUGGA-3'
rno-miR-20a-5p	5'-UAAAGUGCUUAUAGUGCAGGUAG-3'
rno-miR-203	5'-GUGAAAUGUUUAGGACCACUAG-3'
rno-miR-204-5p	5'-UUCCCUUUGUCAUCCUAUGCCU-3'
rno-miR-215	5'-AUGACCUAUGAAUUGACAGAC-3'
rno-miR-25-3p	5'-CAUUGCACUUGUCUCGGUCUGA-3'
rno-miR-29b	5-'UAGCACCAUUUGAAAUCAGUGUU-3'
rno-miR-92a-3p	5'-UAUUGCACUUGUCCCGGCCUG-3'
rno-miR-93-5p	5'-CAAAGUGCUGUUCGUGCAGGUAG-3'

References:

1. Ku GM, Kim H, Vaughn IW, Hangauer MJ, Myung Oh C, German MS, and McManus MT. Research resource: RNA-Seq reveals unique features of the pancreatic beta-cell transcriptome. Molecular endocrinology 26, 1783-92 (2012).
